# Theory of the Origin, Evolution, and Nature of Life 

**DOI:** 10.3390/life2010001

**Published:** 2011-12-23

**Authors:** Erik D. Andrulis

**Affiliations:** Department of Molecular Biology and Microbiology, Case Western Reserve University School of Medicine, Wood Building, W212, Cleveland, OH 44106, USA; E-Mail: exa32@case.edu; Tel.: +1-216-368-0261; Fax: +1-216-368-3055

**Keywords:** quantum, gyre, emergence, thermodynamics, singularity, natural law, adaptation, learning and memory

## Abstract

Life is an inordinately complex unsolved puzzle. Despite significant theoretical progress, experimental anomalies, paradoxes, and enigmas have revealed paradigmatic limitations. Thus, the advancement of scientific understanding requires new models that resolve fundamental problems. Here, I present a theoretical framework that economically fits evidence accumulated from examinations of life. This theory is based upon a straightforward and non-mathematical core model and proposes unique yet empirically consistent explanations for major phenomena including, but not limited to, quantum gravity, phase transitions of water, why living systems are predominantly CHNOPS (carbon, hydrogen, nitrogen, oxygen, phosphorus, and sulfur), homochirality of sugars and amino acids, homeoviscous adaptation, triplet code, and DNA mutations. The theoretical framework unifies the macrocosmic and microcosmic realms, validates predicted laws of nature, and solves the puzzle of the origin and evolution of cellular life in the universe.

## 1. Introduction

How life abides by the second law of thermodynamics yet evolutionarily complexifies and maintains its intrinsic order is a fundamental mystery in physics, chemistry, and biology [1]. Solving this problem requires an interdisciplinary knowledge and an awareness of conventional theories, especially those related to the origin and evolution of life. Rather than give a comprehensive literature review, I introduce a handful of these ideas and point out their limitations.

The panspermia hypothesis has many forms, some of which suggest that life started elsewhere in the universe and arrived on Earth by cometary, meteoric, or planetary delivery [2,3]. The problem with this group of models is that it does not, in an empirically complete and consistent manner, explain the molecular origin of the first cell and hence avoids the issue in need of solution. The primordial soup hypothesis, also know as the Oparin-Haldane model, posits that during the early evolution of the Earth, a reducing atmosphere provided the correct environment for the formation of basic organic compounds [4,5]. Though the soup model has matured in recent decades, it has difficulty explaining the exact conditions of the early Earth atmosphere and the manner and order of emergence of polymeric systems. In the iron-sulfur world theory, primitive life is assumed to have started at deep-sea hydrothermal vents as a mineral base; redox reactions provided the chemical energy to drive the emergence of cellular life [6]. However, this model does not explain the origin of genetic information, membrane systems, or the complexification or diversity of cellular structure. Finally, the RNA (ribonucleic acid) world hypothesis posits that ribonucleotide-based genetic systems evolved prior to protein and deoxyribonucleic acid (DNA). This hypothesis does not fit well with the central dogma and is unable to resolve precisely how the translation apparatus, genetic code, and biometabolic pathways evolved [7,8,9]. In short, no consensus model for life has emerged.

Now, therefore, to know what life is and how life works, scientists need a scientifically accurate theory. The aim of a scientific theory is to construct a formal structure—in which the natural world is being modeled—to explain, predict, and control systems, events, and objects. Insofar as the physical, chemical, and biological sciences are *true*, physical reality and life itself thus reflexively model such a scientific theory; tautologically, the natural world subsumes said theory. Several investigators have detailed what would be required of a unifying bioscientific theory [1,10,11,12,13,14,15,16,17,18,19,20,21,22,23,24]. The correct theory would be expected to not only explain how the living cell works *now*, but also to provide insight into the evolution of life on Earth.

In the theory proposed herein, I use the heterodox yet simple ***gyre***—a spiral, vortex, whorl, or similar circular pattern—as a core model for understanding life. Because many elements of the gyre model (***gyromodel***) are alien, I introduce neologisms and important terms in bold italics to identify them; a theoretical lexicon is presented in Table 1. The central idea of this theory is that all physical reality, stretching from the so-called inanimate into the animate realm and from micro- to meso- to macrocosmic scales, can be interpreted and modeled as manifestations of a single geometric entity, the gyre. This entity is attractive because it has life-like characteristics, undergoes morphogenesis, and is responsive to environmental conditions. The gyromodel depicts the spatiotemporal behavior and properties of elementary particles, celestial bodies, atoms, chemicals, molecules, and systems as quantized packets of information, energy, and/or matter that oscillate between excited and ground states around a singularity. The singularity, in turn, modulates these states by alternating attractive and repulsive forces. The singularity itself is modeled as a gyre, thus evincing a thermodynamic, fractal, and nested organization of the gyromodel. In fitting the scientific evidence from quantum gravity to cell division, this theory arrives at an understanding of life that questions traditional beliefs and definitions.

**Table 1:** Gyromodel Lexicon ^a^.

Term	Meaning
Alternagyre	A gyrosystem whose gyrapex *is not* triquantal
Dextragyre	A right-handed gyre or gyromodel
Focagyre	A gyre that is the focal point of analysis or discussion
Gyradaptor	The gyre singularity—a quantum—that exerts all forces on the gyrosystem
Gyrapex	The relativistically high potential, excited, unstable, learning state of a particle
Gyraxiom	A fact, condition, principle, or rule that constrains and defines the theoretical framework
Gyre	The spacetime shape or path of a particle or group of particles; a quantum
Gyrequation	Shorthand notation for analysis, discussion, and understanding gyromodels
Gyrobase	The relativistically low potential, ground, stable, memory state of a particle
Gyrognosis	The thermodynamically demanding process of learning and integrating IEM
Gyrolink	The mIEM particle that links two gyromodules in a gyronexus
Gyromnemesis	The thermodynamically conserving process of remembering and recovering IEM
Gyromodel	The core model undergirding the theoretical framework
Gyromodule	A dIEM particle in a gyronexus
Gyronexus	A polymer of dIEM particles linked by mIEM particles
Gyrostate	The potential and/or kinetic state that a particle occupies in its gyratory path
Gyrosystem	A gyromodel with specific IEM composition, organization, and purpose
IEM ^b^	Information, energy, and/or matter
Levoragyre	A left-handed gyre or gyromodel
Majorgyre	A gyrosystem whose gyrapex *is* triquantal
Matrioshkagyre	A model that demonstrates how gyres organize in nested sets
Ohiogyre	Higher-order organization in which a gyre gyrates around another gyre
Particle	A discrete, finite, empirically definable unit of IEM
Quantal	Of or relating to the quantum; tri-, di-, uni- and aquantal gyrostates found in majorgyres
Quantum	A capacious, potentially infinite, uncertain unit of IEM; a gyre
Subgyre	The gyre that subsumed by the focagyre
Supragyre	The gyre that subsumes the focagyre
Trimergence	Evolutionary emergence of a triquantal IEM
**Prefixes** ^c^	
Amino	Of or relating to sulfur compounds (particles), amino acids, polypeptides
Carbo	Of or relating to carbon particles, carbohydrates, hydrocarbons
Cellulo	Of or relating to cells, archaebacteria, eubacteria, eukaryotes
Electro	Of or relating to visible matter particles, chemical elements, planetary cores
Geno	Of or relating to genes, DNA, chromosomes, genomes
Oxy	Of or relating to oxygen particles, water, oceans, lunar cores
Phospho	Of or relating to phosphate particles, phospholipids, phosphate signaling
Ribo	Of or relating to nitrogen particles, nitrogenous bases, RNA
**Suffixes** ^c^	
–cycle	The spacetime period to complete a regular series of events in the same order
–gyre	Having the quality of a vortex; characterized by cyclical, oscillatory, and unpredictable motion; attractorepulsive, expansocontractive, and creatodestructive
–gnose	Characterized by learning or by IEM consideration and integration
–helix	Having a three-dimensional twisting, winding shape like that of a spiral staircase
–matrix	Having a three-dimensional networked, latticed shape like graphene or an ice crystal
–mneme	Characterized by memory or by IEM storage and retrieval
–nexus	Being connected or linked in a series
–on	Having the quality of a quantum; a particle or an amalgam of such particles
–sphere	Having orb-like features and hyperbolic geometry

^a^ This lexicon is presented alphabetically. In several circumstances, this ordering of words causes definitional cascading—that is, reading of word 1 uncovers an undefined word 2; reading the definition of word 2 reveals undefined word 3; the definition of word 3 provides an ultimate explanation and a meaningful backdrop for understanding words 1 and 2. ^b^ The gyromodel has defining IEM (dIEM) and modifying IEM (mIEM) particles. ^c^ Each prefix is combined with each and every suffix to expand the lexicon of the theoretical framework. This neologistical appending reveals the commonality between, within, and among the distinct gyrosystems.

## 2. Model

Throughout history, scholars have used the gyre in their models. For example, in ancient Greece, Democritus posited vortex motion to be a law of nature. In the 16^th^ century, Copernicus modeled planets gyrating around a stellar singularity and Descartes proposed his vortex theory for planetary motion in the 17^th^ century. The 19^th^ century found Helmholtz rediscovering the Democritean law and Lord Kelvin and Maxwell using the gyre as the basis of different electromagnetic theories. In the early 20^th^ century, Bostick used the gyre in his spiraling helicon fiber model and Thomson proposed that atoms were vortex rings. Many others have promulgated the gyre as core model of nature.

Perhaps one reason for their theoretical appeal is that gyres are detectable throughout the cosmic and tellurian realms. Astronomically, galaxies, solar systems, comets, and lunar bodies gyrate. Atmospherically, tornadoes, hurricanes, eddies, and vortex streets are all gyres. Oceanographically, there are seven major gyres. Molecularly, numerous nucleic acid and protein structures—DNA double helix, RNA hairpins, pseudoknots, α-helices, coiled coils, and β-propellers—all gyrate. Cellularly and organismally, shells, horns, antennae, flagellae, and the cochlea all carry a spiral imprint. Given its theoretical pedigree, empirical ubiquity, and dynamic character, the gyre appears, *a posteriori*, to be a prime candidate for a core model of natural systems.

### 2.1. Gyre Facts

There are numerous facts that characterize all gyres [25,26,27,28]. These facts—introduced here for propaedeutic purposes—demonstrate that the gyre is protean. For this presentation, I have separated these facts into four broad, overlapping categories and subsections: gyre structure, gyre qualities, gyre thermodynamics, and gyre forces. I conclude this section with a brief summary regarding the gyre and its relevance to theoretical pursuits.

#### 2.1.1. Gyre Structure

A visual examination of the gyre reveals a remarkably plastic geometric form. That is, gyres manifest particular shapes and patterns of a non-Euclidean form. Viewed transversely, many gyres are elongated, helicoid, conical, or funnel-shaped, with a tapered bottom that ends in a point or singularity and have a wide aperture at their top. Other gyres are cylindrical, catenoid, flattened, or disc-like. When viewed head on, both the singularity and aperture frequently appear as perfect circles, like in a galactic center or the eye of a hurricane. Measurements from the singularity of a natural gyre to its circumferential aperture show exponential growth whereas the converse shows exponential decay. Any gyre is fractal because of its self-similarity, fine structure, and simple and recursive nature.

The gyre singularity is defined here as the central position around which energy and matter (discussed further in 2.3.1) revolve. The singularity is also the point of highest energy and matter density and potency in the gyre. Suggestive of the applicability of the gyre to modeling nature, the singularity concept is found both in astrophysics [29,30] and in life sciences [31]. Gyres are also symmetrical: they have organizational or compositional reflectivity, identity, or similarity around a radial axis that bisects the singularity. This symmetry is detectable in spiral galaxies, chemicals like heme, and macromolecules structures like the centrosome.

**Figure 1:**
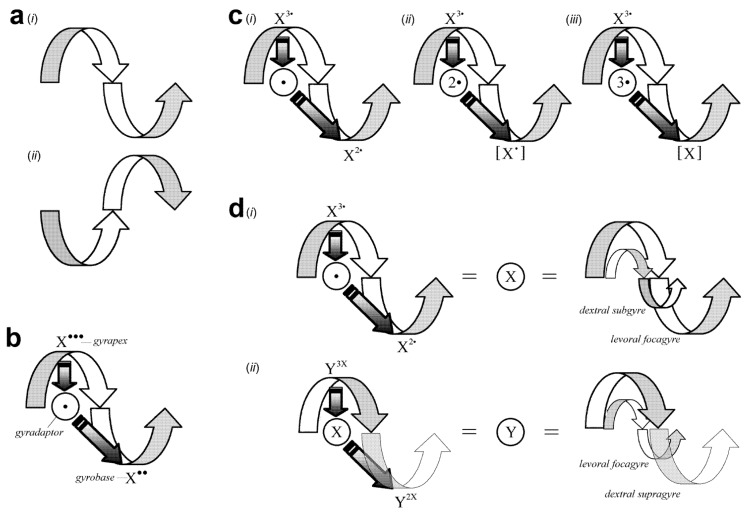
Core theoretical concepts. (**a**) Gyromodel chirality. (*i*) Transverse view of a left-handed gyre (levoragyre). (*ii*) Transverse view of a right-handed gyre (dextragyre). The first and second half-turns of the gyres are depicted as bent arrows. White, gyre interior; grey, gyre exterior. (**b**) Archetypal gyromodel. This gyromodel—supplemented with symbolic variables—is an exemplar for understanding IEM emergence, adaptation, movement, and evolution in the natural world. The bold straight arrows represent IEM directionality. The first bold arrow, from the gyrapex (X^•••^) to the gyradaptor (ʘ), represents mIEM particle (•) attraction (absorption) to the singularity, causing the diquantal dIEM (X^••^) to cycle to the gyrobase. The second bold arrow, from the gyradaptor to the gyrobase, represents the mIEM particle repelled (emitted) from the singularity, ultimately causing the diquantal dIEM to cycle to the gyrapex, restoring the triquantal dIEM (next cycle not shown here). The gyromodel thus depicts an *open* thermodynamic system. (**c**) Majorgyres. Majorgyres are the three main gyromodels at the core of each gyrosystem in the theoretical framework: (*i*) primary (1°) majorgyre; (*ii*) secondary (2°) majorgyre; and (*iii*) tertiary (3°) majorgyre. Note how the gyrapex is shared by all three majorgyres. (**d**) Gyre-quantum equivalence and Matrioshkagyres. *Left-side equations*. (*i*) The gyre—modeling the cycling • on/in and off/out of X due to the attractorepulsive quantum ʘ—is the compressed into Ⓧ, a quantum. (*ii*) Ⓧ, in turn, is the gyradaptive force responsible for cycling X on/in and off/out of Y. *Right-side equations*. (*i*) The ʘ is a dextral subgyre (dextrasubgyre) within the levorafocagyre. (*ii*) The levorafocagyre, in turn, is antichiral to the dextrasupragyre. Ⓧ and Ⓨ are thus both antichiral Matrioshkagyres.

Gyres are chiral, *i.e*., have handedness. When viewed head on, a left-handed gyre rotates clockwise; a right-handed gyre rotates counter-clockwise. The paradox of chirality is that a left-handed gyre, when inverted 180° and viewed anew, is a right-handed gyre. This paradox is at the core of the problem of life. Indeed, homochirality—exclusive use of one chiral form or the other—is observed throughout life, where sugars are dextral (D), amino acids in polypeptides are levoral (L) and nucleotides in nucleic acids are D form [32]. With this paradox in mind, the core, generic gyromodel can be viewed as either left-handed (***levoragyre***; (Figure 1a (*i*)) or right-handed (***dextragyre***; (Figure 1a (*ii*)).

#### 2.1.2. Gyre Qualities

There are several characteristics of a gyre that make it theoretically appealing. Most notably, gyres are organic, that is, they have qualities identical to those found in living systems: they adapt their shape, size, position, rate, strength, and direction. Furthermore, gyres follow a life cycle of emergence (birth), development (aging), and dissolution (death). Gyres spontaneously self-organize when the pressure, temperature, energy, and matter conditions are appropriate. Foreshadowing gyromodel application, scientists have proposed that the universe, matter, molecules, cells, and ecosystems, among other aspects of nature, are self-organizing [33,34,35,36]. Given gyre spontaneity, the precise spatiotemporal coordinates of gyre emergence or trajectory are unpredictable. Likewise, accurately predicting gyre strength and composition is beyond current scientific techniques.

This unpredictability is found in nonlinear equations: gyres do not operate in a sequential or deterministic manner and therefore do not permit simple mathematical depiction. Restated, the versatile gyre does not avail itself to the predictive power of mathematics. As an aside, it is worth mentioning that a complete and consistent mathematical model of the universe is thought impossible due to Gödel’s incompleteness [37,38]. The vicissitudinous gyre, though non-mathematical, epitomizes nature.

#### 2.1.3. Gyre Thermodynamics

Gyres are open thermodynamic entities that require energy and matter mobilization to establish and maintain themselves. Being open systems, gyres import energy and matter from their surroundings into themselves, ebb and flow energy and matter within themselves, and dissipate energy and matter from themselves into their surroundings. Reducing or increasing amounts of energy and matter elicits gyre contraction or expansion, respectively. When efflux or influx is acute, extreme, or unsustainable, a gyre collapses. A gyre staves off collapse through autoregulation: a gyre feeds into itself, regulating its own rotational rate, size, composition, motion, and trajectory. Gyre autoregulation is spatiotemporally internal and/or external, proximal and/or distal, negative and/or positive. Consistent with its autoregulatory bent, a gyre maintains homeostasis—internal responsiveness and balance—by oscillating material around its singularity, a consequence of alternating between extreme countervailing forces within itself (2.1.4). Previewing the application of the gyromodel to life, the cell is an open thermodynamic entity that has numerous, discrete layers of autoregulation [39,40,41].

When considering the directionality of ebb and flow of a gyre, a careful examination reveals that—in the absence of physical blockage or inhibition—energy and matter at a spacetime point has *potential* to move omnidirectionally from that point. When this potential is actualized for an excessively large number of matter particles, the geometric form created is called a hypersphere; this shape is compatible with ideas regarding the thermodynamic expansion of the universe [42,43]. Still, in nature, there is manifest directionality, such as that observed in the N- to C-terminal orientation of the protein chain or 5’ to 3’ orientation of nucleotide polymers. Though gyromodels are depicted as having a left-to-right vectorization (Figure 1a), this is simply a two-dimensional restriction of the artistic approach. From this two-dimensional perspective, one revolution of a gyre is seen as a circle or oval. A circle, when viewed in three dimensions, is a cycle. Closing this circle of thought: a cycle viewed in the context of time, or four dimensions, looks like a rotating spiral, helix, or gyre. Foreshadowing, *any* cycle that exists in nature—in physical, chemical, or biological systems—may be viewed as a gyre.

#### 2.1.4. Gyre Forces

All natural gyres harbor two countervailing forces: attraction and repulsion. Paradoxically, the gyre singularity both attracts *and* repels energy and matter and thus is “attractorepulsive.” These unified yet contradictorily dual (diune) forces exert paradoxical effects. For example, individually, the attractive and repulsive forces can elicit *both* aggregation *and* dis-aggregation of energy and matter; these creative and destructive (“creatodestructive”) effects are relative to the singularity. Moreover, a gyre can undergo expansion *or* contraction (“expansocontractive”) as a consequence of energy and matter influx or efflux. Taken together, these countervailing forces make for a matrix of diune or multiple phenomena (e.g., “attractocontractive,” “repulsocreative,” “expansodestructive,” and so on). Gyre forces occur both within an individual gyre and also between and among gyres. For example, two transverse gyres exhibit constructive interference when synchiral (same chirality) and destructive interference when antichiral (opposing chirality). Alternatively, the fine-tuning and balancing of two contradictory forces results in neutrality, immutability, and immobility—identifiable characteristics of physical systems. I detail these forces as they relate to the gyromodel in 2.4.6.

#### 2.1.5. Gyre Summary

The prior sections demonstrate that the gyre is a basic and concrete model of broad applicability, a profound heuristic, and an unchanging form that changes. Further, the gyre evidently fulfills many of the modeling requirements of complexity, emergence, chaos, systems, information, and evolutionary theory [44,45,46,47,48,49,50,51]. As such, the burden of proof for the gyre as the core model of nature is heavy; this manuscript represents the deposition of empirical testimony in theoretical court.

### 2.2. Gyromodel Caveats

Life is complex and perplexing. It should come as no surprise that modeling life is a complicated procedure. Likewise, explaining a theory of life is an arduous task. Thus, prior to proceeding, I issue several warnings regarding the model and theory.

The gyromodel is incommensurable with prior and existing theories. Thus, the reader must judge this theory by two criteria: the principle of parsimony, or Ockham’s Razor [52]—the scientific principle dictating that things behave or are connected in the simplest and most economical fashion—and the ability to explain the available scientific data. Another challenge is discovered in the lexicon, where I have redefined established terms and created and applied ~100 new words to identify, explain, and interconnect distinct aspects of the theory (Table 1). Creating a new vocabulary yields, on the one hand, a single, tight system to unify multiple disparate scientific languages. On the other hand, simultaneously supplanting the vernaculars of physics, chemistry, and biology may cause a high degree of frustration. Together, the foreign symbolism, semantics, and lexicon make comprehending the gyromodel difficult. As more *is* different [53], one must think differently to interpret more. Finally, this theory challenges long-held assumptions, guiding philosophies, *ad hoc* models, cherished paradigms, ossified boundaries, and, quite regrettably, patience.

These warnings represent a full and sincere disclosure of the difficulties in effectively presenting my model and theory and of convincing the reader of its scientific merit. I also mean to emphasize, up front, that this manuscript is dense.

### 2.3. Gyromodel Organization

I now begin the technical overview of the gyromodel in earnest. This section is presented in four subsections. The first defines information, energy, and matter and foreshadows how these three elements integrate throughout the theory. The second part establishes a gyromodel-specific relationship between a physical “particle” and a “quantum.” The third part deals with two specific states that are modeled onto the gyre. The fourth and final part introduces three major gyromodel forms and applies a familiar notation to explore their explanatory utility. By laying out the core template and its rules in entirety prior to data fitting, I ensure that everything that is not prohibited is required.

#### 2.3.1. Information, Energy, and Matter

The three main components that are represented by, stored in, and mobilized within, between, and among gyres are information, energy, and/or matter (***IEM***): Information is theoretically defined as something that conveys and harbors meaning; energy is something that is used to perform work; and matter is something that occupies space and has mass [54]. There are several points regarding IEM that require development. For instance, an object or a system can possess different types of energy, including but not limited to potential, kinetic, electromagnetic, chemical, and heat. Life is replete with examples of these different types: endo- and exothermic (energy input and energy output, respectively) biophysical processes, biochemical pathways, biogeochemical cycles, and inorganic chemical reactions [55,56,57]. Moreover, energy and matter are interconvertible based upon their mathematical equivalence (E = mc^2^): energy can be transformed into matter and mass into energy. Information, in turn, is the distinct patterns or organizations of energy and matter, with these patterns detectable by observation and quale [58]. Even though certain modeled objects or systems could be considered as largely one of these three components, I use the acronym IEM throughout the text to denote the composite nature and interrelatedness of information, energy, and matter.

I integrate IEM into the gyromodel as symbols. These symbols are variables, representing one or more gyre components or set(s) of components. Although these symbols (components) are written adjacent to the gyre, they are the gyre itself. Throughout this exposition, I highlight important notations by offsetting them from the text.

#### 2.3.2. Particle and Quantum

The generic gyromodel is decorated with several symbols (Figure 1b). For example, ʘ denotes the gyre singularity. For the compound symbols X^•••^ and X^••^, X is called the *defining* IEM (***dIEM***) and • is *modifying* IEM (***mIEM***) of the gyre. Whereas the dIEM typifies a gyre system (***gyrosystem***)—there are several unique systems that require modeling—the mIEM changes the quality, content, or application of the dIEM. Any single (X or •) or compound (X^•••^ or X^••^) component is called a ***particle***. For theoretical thrift, X^•••^ or X^••^ represents either one particle or many particles of similar composition.

The singularity, ʘ, models an IEM packet called a ***quantum***. The quantum exerts an adaptive force on the gyrosystem (and thus is termed the ***gyradaptor***), modulating the thermodynamic and spatiotemporal properties of particles. The quantum captures the infinite IEM potential:

• + ʘ = ʘ (*i.e*., any number + ∞ = ∞)

In the gyromodel, there is an inverse conceptual relationship between a particle and a quantum: Though the quantum typically represents *all* germane IEM, it can represent one, this being the particle; though a particle typically represents *one* germane IEM, it can represent all, this being the quantum.

#### 2.3.3. Gyre States

The generic gyromodel appears to show two particles, one occupying each gyre half-turn (Figure 1b). However, this is not *two* dIEM particles, but rather *one* dIEM particle that gyrates from one distinct, extreme spacetime state to another (***gyrostates***). The excited state (X^•••^) is the apex of the gyre, the ***gyrapex***. The ground state (X^••^) is the base of the gyre, the ***gyrobase***. dIEM oscillation between gyrostates occurs *via* particle exchange to and from the gyradaptor. The mIEM particle, by comparison, can exist in only one of three spacetime coordinates: the gyrapex, gyrobase, or gyradaptor. Given gyrostate oscillation, the gyromodel accords with energetic coupling such as that seen in biometabolism.

#### 2.3.4. Majorgyres

Gyromodels with a ***triquantal*** (where ••• = 3^•^) gyrapex and either a di- (•• = 2^•^), uni- (•), or aquantal gyrobase define the three ***majorgyres***—the major gyromodels of the theoretical framework (Figure 1c). The gyrapex and ***primary majorgyre*** (1°; Figure 1c (*i*)) have already been discussed as in the context of the generic gyromodel (2.3.2; 2.3.3; Figure 1b).

For the ***secondary majorgyre*** (2°; Figure 1c (*ii*)), the gyradaptor is 2ʘ, a diquantum, and the gyrobase, [X^•^], where [ ] represents a unit that has the *potential* to multimerize, is:

X^•^ (monomer), X^•^X^•^ (dimer), X^•^X^•^X^•^ (trimer), and/or X^•^X^•^X^•^X^•^X^•^X^•^X^•^…(polymers)

Note that the mIEM (•) “links” the dIEM (X) in the polymer; I expand upon the linkage concept below (2.4.3). In the ***tertiary majorgyre*** (3°; Figure 1c (*iii*)), the gyradaptor is 3ʘ, a triquantum, and the gyrobase, [X], is:

X, XX, XXX, and/or XXXXXX….

Here, an IEM inherent to X itself is the quantal “link” (not shown to maintain modeling consistency). When [X^•^] and [X] are two or more units, they are a particle nexus termed a ***gyronexus***.

An accessible way of presenting these majorgyres is by reaction equation shorthand (***gyrequations***):

Primary majorgyre: X^3•^ ⇆ X^2•^ + ʘ

Secondary majorgyre: X^3•^ ⇆ [X^•^] + 2ʘ

Tertiary majorgyre: X^3•^ ⇆ [X] + 3ʘ

There are additional features of the gyromodel that can be elaborated using gyrequations. A majorgyre can be multiplicative, for example:

(X^3•^ ⇆ X^2•^+ ʘ)_n_,

where n = any positive integer. These multiples, in turn, can undergo division or fractalization. Further, using this notation, majorgyres can be balanced like chemical reactions. Since X^3•^ is found in both primary and secondary majorgyres, these can be rewritten as:

X^2•^+ ʘ ⇆ X^3•^ ⇆ [X^•^] + 2ʘ

Removing the X^3•^ intermediate, thus compressing the gyrequation, reveals:

X^2•^+ ʘ ⇆ [X^•^] + 2ʘ

And balancing the gyrequation by subtracting out ʘ from both sides gives:

X^2•^ ⇆ [X^•^] + ʘ

another pair of particle gyrostates; this represents one of many alternative gyre forms (***alternagyres***).

While a particle can be described by gyrequations in practice, it must be considered as part of larger gyre within which it resides in theory. By extension, a gyre must be considered in the context of its gyrosystem within which it exists. Despite their complexity, gyrosystems that share IEM can be multiplied, divided, added, or subtracted, allowing modeling of multi-component systems with gyrequations. Although a gyrequation excludes the vectorial and adaptive nature of IEM flow, it is a compact and tractable notation. Further, the gyrequation reminds of chemical equations that symbolically represent chemical reactions. Based upon this familiar and standardized format, I use gyrequations to substantiate and extend upon the gyromodels.

### 2.4. Gyromodel Fundamentals

The foremost purpose of this subsection is to introduce several fundamental features of the gyromodel. A secondary purpose is to give certain words—associated with nebulous, misunderstood, or complex concepts or phenomena—a model-specific meaning. I have organized this subsection into six parts. In the first, I explain the relationship between a gyre and a quantum. In the second, I discuss how the gyromodel treats complementary wave and particle forms. Third, I expand upon the quantal “link.” Fourth, I model learning and memory onto the two gyrostates. Fifth, I introduce the concept of gyrosystem relativism. Finally, I close with a passage that explores gyromodel dynamics.

#### 2.4.1. Gyre-Quantum Equality

In the gyromodel, a gyre is equivalent to a quantum (Figure 1d (*i*), left side equations):

(X^3•^ ⇆ X^2•^ + ʘ) = Ⓧ

As shown, Ⓧ captures the full range of potentialities for the primary majorgyre. Importantly, however, Ⓧ is variable, representing *any* majorgyre or alternagyre. Given gyre-quantum equivalence, then, Ⓧ models a gyradaptor that cycles mIEM particles (X) through dIEM particles (Y) of a supervenient gyre (Figure 1d (*ii*), left side equations), which is itself a quantum, Ⓨ.

For orientation, the gyre/quantum that is the focal point of analysis is the ***focagyre*** (focaquantum); the gyre/quantum subsumed by the focagyre is called a ***subgyre*** (subquantum); and that which subsumes the focagyre is called a ***supragyre*** (supraquantum). Thus, ʘ is a subgyre, Ⓧ is a focagyre, and Ⓨ a supragyre. IEM thermodynamics are unidirectionally simplified as follows:

→ ʘ → Ⓧ → Ⓨ →

The arrows that flank the modeled gyrosystems depict the excluded intrinsic and extrinsic gyres; feedforward and feedback are also excluded.

Because gyre-quantum equality may elicit cognitive dissonance, it is useful to reify this concept by replacing the quantal form with the gyre form (Figure 1d (*i*) and (*ii*), right side equations): ʘ becomes a dextral subgyre within Ⓧ, a levoral focagyre that is within Ⓨ, the dextral supragyre. As this nested antichiral gyre organization is similar to that found in Matrioshka dolls, these models are called ***Matrioshkagyres***. The countervailing forces exerted by antichiral Matrioshkagyres impart gyroscopic stability. Matrioshkagyres can also be synchiral. However, the greater the IEM flow rate, potency, and amount, the higher the probability that a synchiral Matrioshkagyre will become imbalanced, torsionally stressed, and collapse. Thus, oscillating chirality of gyres is essential for gyre maintenance and propagation.

Modeling the Matrioshkagyre in a gyrequation, the supragyre is

Y^3X^ ⇆ Y^2X^ + Ⓧ

where, given gyre-quantum equivalence,

Ⓧ = X^3•^ ⇆ X^2•^ + ʘ

then:

Y^3X^ ⇆ Y^2X^ + (X^3•^ ⇆ X^2•^ + ʘ)

For the sake of this propaedeutic, let

ʘ = •^3Z^ ⇆ •^2Z^ + Ⓩ

then, substituting again, I have:

Y^3X^ ⇆ Y^2X^ + (X^3•^ ⇆ X^2•^ + (•^3Z^ ⇆ •^2Z^ + Ⓩ))

This schema captures the nested relationship among primary majorgyrosystems, the inherent variability of each, and how one cannot be studied independently without loss of IEM of another. Complexifying further, given the inverse quantum-particle relationship and gyre-quantum equivalence, each mIEM can model as a gyrosystem as well:

Y^3(X3• ⇆ X2• + (•3Z ⇆ •2Z + Ⓩ))^ ⇆

Y^2(X3• ⇆ X2• + (•3Z ⇆ •2Z + Ⓩ))^ + (X^3(•3Z ⇆ •2Z + Ⓩ)^ ⇆ X^2(•3Z ⇆ •2Z + Ⓩ)^ + (•^3Z^ ⇆ •^2Z^ + Ⓩ)).

Because Matrioshkagyres and gyrosystems are continually adapting and can be any combinations of major- or alternagyres, the fractal depth of any gyrequation is infinite.

#### 2.4.2. Wave-Particle Unity

The gyromodel clarifies how a quantum has both wave and particle qualities: as one particle oscillates between two extreme gyrostates, its gyratory path creates an undulating pattern that is detected as a wave. When many particles oscillate around the same or different singularities, they create constructive or destructive waveforms. When the gyromodel is considered as a gyre, it manifests classical wave characteristics: wavelength, amplitude, and frequency. When considered as a quantum, it exhibits particle characteristics: translational, rotational, and vibrational movement. The gyromodel thus accounts for particle spin.

#### 2.4.3. Gyronexus Links and Modules

With gyre-quantum equivalence and quantum-particle relations disclosed, I am now able to expand upon the gyronexus “link” as was described for the secondary and tertiary majorgyres (2.3.4). Reviewing, a gyronexus is composed of two parts: the dIEM particle that is being linked (called here a ***gyromodule***), and the mIEM particle that links (a ***gyrolink***). When modeling the gyronexus in a secondary majorgyre, the gyrolink that is a subgyre mIEM has antichiral spin to the gyromodular dIEM. For the tertiary majorgyre, the gyrolink that is a sub_2_gyre (the gyre within the subgyre) mIEM has synchiral spin to the gyromodular dIEM.

In addition to envisioning the dIEM and mIEM as spinning particles, one must also think of higher-order rotations, or orbits, of one particle around another—*i.e*., a gyrolink spinning on its own axis while simultaneously orbiting an axially rotating gyromodule. Hence, a gyrolink is dynamic, *not* static. Building upon this dynamism, since every particle is attractorepulsive, one gyrolink particle can mobilize from one opposing gyromodular area to another in a toroidal or plectonemic spacetime path. Examples of polymers that exist in the natural world for which the gyronexus concept applies: oxygen atoms linked by hydrogen atom electrons as found in water; oxygen atoms from water link carbohydrates in polysaccharides; orthophosphate links nucleotides in an RNA chain; and amide groups link amino acids in a polypeptide.

#### 2.4.4. Gyromodel Learning and Memory

In this section, I articulate how the dynamics and interchangeability of information, energy, and matter within a gyre relates to the retention of these things within the gyre itself. In other words, I establish strict *non-cognitive* meanings for learning and memory as they relate to the gyromodel. Learning is a continual, unstable, and energetically demanding affair. Gyre learning, or ***gyrognosis***, is the process by which the gyradaptor repels the particle from the gyrobase to the gyrapex. This is vectorially modeled in the primary majorgyre as:

ʘ + X^••^→ X^•••°^

where •° represents the learned IEM. Gyrognosis also involves the reorganization of the learned IEM in the gyrapex, for example,

X^•••°^→ X^••°•^, X^•°••^, or X^°•••^

Memory, by comparison, is a relativistically stable and energetically conserving phenomenon. The process of storing IEM in gyre memory, or ***gyromnemesis***, is modeled as:

X^•••°^→ ʘ + X^••°^

The ultimate state of gyromnemesis is the stably adapted particle or gyronexus in the gyrobase.A particle thus adapts through learning and memory by completing one full cycle—a revolution—around the singularity. Taken together, gyrognosis defines IEM integration and assessment whereas gyromnemesis defines IEM storage and recovery. Finally, although a diquantal IEM (X^••^) undergoes gyrognosis as the gyrobase of a primary majorgyre, it undergoes gyromnemesis as the gyrapex of an alternagyre. Thus, gyre learning and memory are relative to the gyradaptive singularity.

#### 2.4.5. Gyromodel Relativity

In considering the majorgyre frame, whereas the gyrapex of the three majorgyres is always the same, the gyrobase and gyradaptor of each is different (Figure 1c). These differences should be considered relativistically. For example, at least symbolically, the 1° gyrobase has compositionally more IEM than does the 2° gyrobase, and the 2° gyrobase has more than the 3° gyrobase. All other things being equal, the IEM in the gyrobases (di-, uni-, and aquantal states) of majorgyres could be written relativistically:

1° > 2° > 3°

In comparison, all things being equal, the IEM in the gyradaptors of the majorgyres is, relativistically:

3° > 2° > 1°

These three aspects of the gyromodel (or any other triad, *i.e*., triquantal, diquantal, and uniquantal forms) should be viewed as follows: (*i*) a high energy (exergic), unstable, excited form; (*ii*) an intermediate energy, quasi-stable, transition form; and (*iii*) a low energy, stable, ground form. Note the antiparallel relationship between gyrobases and gyradaptors, where the unstable gyrobase is accompanied by the stable gyradaptor, and *vice versa*. However, given that many disparate and dynamic IEM species need to be incorporated, gyromodel relativity is ever changing.

#### 2.4.6. Gyromodel Dynamics

Here, I elaborate on gyromodel forces, directionality, chirality and collapse. As the Matrioshkagyre (Figure 1d) models, the singularity of a gyre is a gyre itself. In any given Matrioshkgyre, then, the attractive and repulsive forces of the supragyre are inherent, a consequence of the attraction and repulsion of the focagyre, which, in turn, are a consequence of the attraction and repulsion of the subgyre; and so on. So, then, while a specific force by or on a particle within a given gyromodel—attraction, repulsion, expansion, contraction, creation, destruction—is albeit separable in principle, it needs to be considered in the fuller, nested context of other forces. Adumbrating an application of the gyromodel in biology, the catabolism (destruction, consumption) of glucose in glycolysis can be and is studied as a linear process and in isolation. However, this process is fundamentally dependent upon——and thus inseparable from—glucose anabolism (creation, production), both in the cell and during nonlinear evolutionary emergence.

In addition to forces *within* a gyromodel, forces are exerted *between* and *among* gyromodels. On this matter, gyromodel orientation and IEM composition impacts ebb and flow. When two parallel synchiral shared-IEM gyromodels (though similar, called here α and β for distinguishability) juxtapose, the α-singularity attracts β-dIEM and –mIEM particles and β–singularity attracts α-dIEM and –mIEM particles, thereupon coalescing into a single, larger gyromodel. Such natural concrescence can be observed, for example, when two waterspouts merge or when two oil droplets fuse. By comparison, the singularities of two antiparallel synchiral shared-IEM gyrosystems attract one another to create a shared singularity and counter-mobilized IEM; this arrangement can be found in two antiparallel bar magnets (aligned side-by-side such that the N and S poles of one magnet appose, respectively, the S and N poles of the other) or in the DNA double helix. Repulsion between gyres—which models gyrosystem individuation—arises due to opposed directionality (head-to-head or tail-to-tail as opposed to head-to-tail) or of the inability to share IEM (*i.e*., gyromodels cycle or harbor distinct dIEM and mIEM). The repulsive (anti-attractive) effects of opposed directionality can be observed experimentally: when two N poles or two S poles of two bar magnets are apposed or when two 5’ ends or two 3’ ends of DNA oligomers are apposed.

As mentioned in 2.1.3, gyre collapse occurs by two extreme means: overcontraction or overexpansion. When, for a given gyre within a gyrosystem, a triquantal mIEM particle is attracted to the singularity, the dIEM particle loses the thermodynamic support of its gyradaptor (the gyrobase of the 3° majorgyre; Figure 1c (*iii*)). Due to autoregulatory feedback within the gyrosystem, accelerative attraction of the singularity spreads (to all existing 1° and 2° majorgyres and alternagyres) and mIEM extraction collapses the gyrosystem into more exergic subgyres. Overexpansion—due to unrelenting influx of mIEM particles—forces the triquantal mIEM onto the dIEM (the gyrapex of all majorgyres), disallowing cycling between two states, thus freezing and ultimately collapsing the gyrosystem. An example of overcontraction in nature is desertification, where water collapse elicits diminished vegetation in an ecological system [59]. An example of overexpansion is in the life cycle of the slime mold *Dictyostelium discoideum*: the multicellular fruiting body state collapses, releasing single-celled spores [60]. Another example of overexpansion is wave collapse in physical systems [61].

### 2.5. Gyromodel Axioms

The forthcoming data placement onto the gyromodel complies with natural laws and ordering principles. In addition to using fundamental strictures, I codify a specific set of inviolable rules, conditions, and truths that guide and undergird the theoretical framework—thirteen major gyromodel axioms (***gyraxioms***) and several axiomatic corollaries. This section explains the application and relevance of these gyraxioms to the impending gyrosystems. For emphasis, the gyraxioms have been italicized.

*Gyraxiom I (G_I_). A quantum is a gyre.* This precept is based upon the equivalence of these entities as they are verbally defined (2.1 and 2.3.2) and symbolically depicted (Figure 1d).

*G_II_. A gyrating particle is a wave.* As gyromodeled (Figure 1b), the particle discloses the nature of its spacetime trajectory as an undulating waveform (2.4.2).

*G_III_. The quantum is either one particle or many particles. G_III–1_. A particle has quantum potential.* These two axioms, originally introduced in 2.3.2, reveal the flexibility of the gyromodel to incorporate the small and the large, the few and the many.

*G_IV_. A particle cannot be reduced from its gyre without IEM loss.* A gyre is a unified, coherent entity. Any gyre aspect cannot be examined separately without decoherence and loss of contextualized information, energy, and/or matter. *G_IV–1_. A gyre cannot be reduced from its gyrosystem without IEM loss.* As a gyrosystem is composed of and defined by numerous alterna- and majorgyres (*i.e*., quanta), gyre composition, structure, and function changes upon isolation from its gyrosystem (2.3.4).

**Figure 2:**
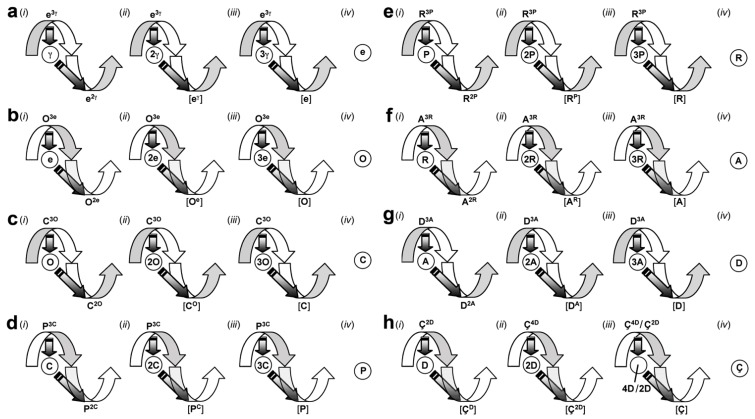
Gyromodels of the theoretical framework. (**a**) Gyromodels of leptonic metabolism. (*i*) 1°, (*ii*) 2°, and (*iii*) 3° electrogyre; (*iv*) electron (e). This quantal form and all subsequent forms represent any of the majorgyres or alternagyres (not shown); γ, photon. (**b**) Gyromodels of oxychemical metabolism. (*i*) 1°, (*ii*) 2°, and (*iii*) 3° oxygyre; (*iv*) oxyon (O). (**c**) Gyromodels of organochemical metabolism. (*i*) 1°, (*ii*) 2°, and (*iii*) 3° carbogyre; (*iv*) carbyon (C). (**d**) Gyromodels of phosphochemical metabolism. (*i*) 1°, (*ii*) 2°, and (*iii*) 3°phosphogyre; (*iv*) phosphon (P). (**e**) Gyromodels of ribonucleotide metabolism. (*i*) 1°, (*ii*) 2°, and (*iii*) 3° ribogyre; (*iv*) ribon (R). (**f**) Gyromodels of amino acid metabolism. (*i*) 1°, (*ii*) 2°, and (*iii*) 3° aminogyre; (*iv*) aminon (A). (**g**) Gyromodels of deoxynucleotide metabolism. (*i*) 1°, (*ii*) 2°, and (*iii*) 3° genogyre; (*iv*) genon (D). (**h**) Gyromodels of cellular metabolism. (*i*) Hapcellulogyre; (*ii*) dipcellulogyre; (*iii*) acellulogyre; (*iv*) cellulon (C). Note the repetitive yet chirally oscillating nature of gyrosystems. This figure complements Table 2.

*G_V_. A particle oscillates between excited and ground states but cannot simultaneously exist in more than one state in spacetime.* A particle transits from one extreme gyrostate to another (Figure 1b,c, and Figure 2; 2.3.3).

*G_VI_. A gyre oscillates between left and right chirality but cannot simultaneously exist as more than one chirality in spacetime.* Relative to an observer, the gyrating trajectory of *one* particle or system can be either clockwise or counter-clockwise, but not both (Figure 1a; 2.1.1, 2.4.1).

*G_VII_. Antichiral Matrioshkagyres are more homeostatic and stable than synchiral Matrioshkagyres.* As introduced in 2.4.1, countervailing gyre chiralities of nested gyrosystems bestows stability on those systems; identical chiralities in nested systems induce runaway gyrosystem acceleration, torque, and disintegration.

*G_VIII_. A focagyre is thermodynamically dependent upon one or more of its subsumed gyres. G_VIII–1_. A focagyre is thermodynamically required for one or more of its supervenient gyres.* Gyrosystem nesting depicts IEM flowing from within to without, from sub- to foca- to supragyre (2.4.1).

*G_IX_. A focagyre contains at least one novel, emergent IEM form distinct from its subgyre.* The omnidirectional expansion and self-organized criticality of the subgyre yields a focagyre with similar organization yet dissimilar composition.

*G_X_. In a secondary majorgyre, the gyrolink of the gyronexus is the dIEM of the subgyre.* The gyrolink of the gyronexus represents the subgyre itself (2.3.4, 2.4.3).

*G_XI_. In the tertiary majorgyre, the gyrolink of a gyronexus is the dIEM of the sub_2_gyre. G_XI–1_. A tertiary majorgyre gyrolink, in coupling to other tertiary majorgyres, facilitates IEM flow between and among subsumed gyrosystems.* These two gyraxioms disclose how long-range IEM thermodynamics and gyrosystem organization occurs (2.3.4, 2.4.3).

*G_XII_. The IEM in primary and secondary majorgyres has subgyre chirality.* In other words, dextral IEM oscillates in a levoral focagyre due to force exerted by dextral subgyre. Levoral IEM oscillates within a dextral focagyre due to force exerted by levoral subgyre (see Figure 1d). *G_XII–1_. When countervailing forces of an antichiral Matrioshkagyre offset exactly, the focagyre IEM does not have chirality.* This axiom provides a basis for a particle without spin or a neutral, illusorily immobile (non-gyrating) state. *G_XII–2_. When considering G_XI_, the IEM in the gyrobase of the tertiary majorgyre has sub_2_gyre chirality. G_XII–3_. Given G_V_ and G_VI_, IEM higher-order organization and fractalization within a focagyre elicits chiral toggling.* As Matrioshkagyres are more stable in an antichiral state, so too complexified IEM within a gyrosystem itself achieves intrinsic balance through countervailing nested chiralities (2.4.3).

*G_XIII_. Subgyres are more exergic and less stable than focagyres.* Relativistically, the subgyre IEM is of a higher quality of energy that is extractable for work. For example, the amount of energy that can be extracted to perform physical work from an electron (*i.e*., to transfer electricity) is greater as compared to the amount of energy extracted to perform physical work from a nucleotide triphosphate (*i.e*., to transfer an orthophosphate bond).

## 3. Theory

I have organized this part into eight subsections, each detailing a discrete, empirically defined system that is amenable to theoretical modeling: visible matter, water, organic matter, phosphomembrane, RNA, protein, DNA, and cell. This theoretical framework concomitantly depicts both the microcosm—the biology, chemistry, and physics of the existing living cell—and the macrocosm—the astrophysical and biogeophysichemical (geospheric, hydrospheric, atmospheric, biospheric) process underlying the evolution of life on Earth. Hence, subdividing this framework into separate parts defined by scale, by field, by topic, or by evolutionary spacetime is not scientifically appropriate for modeling life *in toto*. Given the manuscript format, a full treatment of alternative theories, models, hypotheses, and arguments is unrealistic. Nevertheless, each subsection overviews germane problems and theories, fits data using gyromodels and gyrequations, and concludes with a point regarding macroevolutionary transition from one quantal form to another. To enhance readability, each subsection is suborganized, with subject matter preceded by self-explanatory paragraphic title.

Throughout this section, I point out four theoretical applications: (*i*) to explain phenomena that have been enigmatic or misinterpreted; (*ii*) to model the organization of particles, atoms, molecules, and systems; (*iii*) to position the origin, emergence, and evolution of one thing relative to another; and (*iv*) to predict modes of operation. Each gyrosystem (Figure 2) is given a unique letter identifier and an etymologically obvious terminology (Table 2). Unless stated otherwise, a gyrosystem neologism refers to more than one or all majorgyres and alternagyres.

There are two points to be made about referencing. First, in many cases, I use only one or two references to defend a position or to guide the reader. I regret that many seminal studies are not cited. Second, in the later subsections, I call attention to literature on the evolution and function of life as it relates to eubacteria, archaebacteria, and eukaryotes. However, I often cite only the most general and principal work on one kingdom or another and on one organism or another. Given these circumstances and if warranted, I urge the incisive and demanding reader to pursue deeper investigation of a particular field or topic.

**Table 2:** Gyrosystem organization ^a^.

					Gyrostates ^b^		Chirality ^c^		
Gyre	Quantum	dIEM	mIEM	Gyradaptor	Gyrapices	Gyrobases	Gyre	1°/2° ^e^	3° ^e^
Electrogyre	Electron	e	γ ^d^	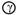	e^3γ^	e^2γ^, [e^γ^], [e]	L	D	L
Oxygyre	Oxyon	O	e	ⓔ	O^3e^	O^2e^, [O^e^], [O]	D	L	D
Carbogyre	Carbyon	C	O	Ⓞ	C^3O^	C^2O^, [C^O^], [C]	L	D	L
Phosphogyre	Phosphon	P	C	Ⓒ	P^3C^	P^2C^, [P^C^], [P]	D	L	D
Ribogyre	Ribon	R	P	Ⓟ	R^3P^	R^2P^, [R^P^], [R]	L	D	L
Aminogyre	Aminon	A	R	Ⓡ	A^3R^	A^2R^, [A^R^], [A]	D	L	D
Genogyre	Genon	D	A	Ⓐ	D^3A^	D^2A^, [D^A^], [D]	L	D	L
Cellulogyre	Cellulon	Ç	D	Ⓓ	Ç^4D^, Ç^2D^	[Ç^2D^], [Ç^D^], [Ç]	D ^f^	L/D ^f^	D/L ^f^

^a^ This table complements the gyromodels in Figure 2. ^b^ Gyrapices are the learning gyrostates; gyrobases are the memory gyrostates. The gyrostates for majorgyres are shown. ^c^ Gyre and IEM exist cosmically in both chiralities but in life are almost exclusively in one chirality (see footnote ‘e’). D, dextral; L, levoral. ^d^ The ‘γ’ models the photon. ^e^ 1°, primary majorgyre IEM; 2°, secondary majorgyre IEM; and 3°, tertiary majorgyre IEM. ^f^ Several gyraxioms—G_VI_, G_VII_, G_VIII_, G_XII_, and G_XIII_—clarify why the gyre and IEM chirality are “primarily” one form in cells as opposed to exclusively one form. As the exergy of the gyrosystem diminishes, such that e >>>>>>> O >>>>>> C >>>>> P >>>> R >>> A >> D > Ç, the rate of IEM flow concomitantly diminishes. The relativistically reduced attractorepulsive effect of the genon on the cellulogyre (compared to earlier gyrosystem relationships) means that the subgyres have greater potential to impact cellulogyre form and function. Thus, while oscillating chirality (G_VI_ and G_XII_) is retained in principle, theory fits the data in practice.

### 3.1. Visible Matter

I begin this theory with the modeling of visible matter, that is, spacetime particles that have mass and can be experimentally manipulated and visualized. I have chosen to start here because, in life, cellular respiration requires an ***electron*** transport chain [62], electron mobilization is fundamental to biophysical assembly and disassembly, biochemical reactions, and signal transduction [63], and, most notably, atomic and quantum models undergird the thinking and experimentation about all cellular molecules [64]. I now turn to modeling life from within to without, from the physics of the quantum to the biology of the cell.

There is overt discussion of a crisis in fundamental physics [65,66]. One reason for this harsh admission is the lack of a solution for the most protracted problem in modern physics: a model that unifies the atomic and cosmic realms [67]. From the early 20^th^ century, there have been two separate models for these two domains. Quantum mechanics (QM) is a mathematical model that describes the physical properties of visible matter [68]. General relativity (GR) is a mathematical theory that describes the universal attractive force, gravity [69]. A unified model of quantum gravity (QG) is expected to explain both the regular dynamics and properties of celestial bodies and all of the well-known quantum properties and enigmas such as spin, wave-particle duality, Heisenberg Uncertainty Principle, and the measurement problem [70]. Such a theoretical marriage would be expected to yield insight into the structure, function, and origin of leptons, chemical elements, planets, and solar systems. In this subsection, I introduce a gyrosystem called the ***electrogyre*** (Figure 2a) and, under the direction of natural laws and gyraxioms, place it onto physical data.

*Lepton*. Microcosmically, the ***primary electrogyre*** (Figure 2a (*i*)) models the single free high-energy electron (**e**) found most commonly in the element hydrogen or any lepton (muon, tau, and neutrinos, each represented by the **e** symbol). Although there are many models of lepton substructure, the exact composition is unclear [71]. As modeled here, the lepton is a visible energy particle (a photon, **γ**) that step-decelerates below light speed due to the opposing, balancing forces and directionalities of the subgyre/subquantum (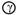; G_VI_, G_VII_, and G_XIII_). In other words, in reducing its vibrational, rotational, and translational rate, the photon particle literally manifests as the lepton particle.

The structure of the primary majorgyre requires a triquantal or diquantal set of photons (triphoton, 3γ, and diphoton, 2γ) to orbit the **e** particle, thus existing in either an excited (e^3γ^; the gyrapex) or ground (e^2γ^; the gyrobase) state in spacetime (G_V_). Oscillation between these two electrogyrostates occurs by virtue of the attractorepulsive, expansocontractive, and creatodestructive forces of the photonic singularity (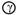; the gyradaptor).

**Figure 3:**
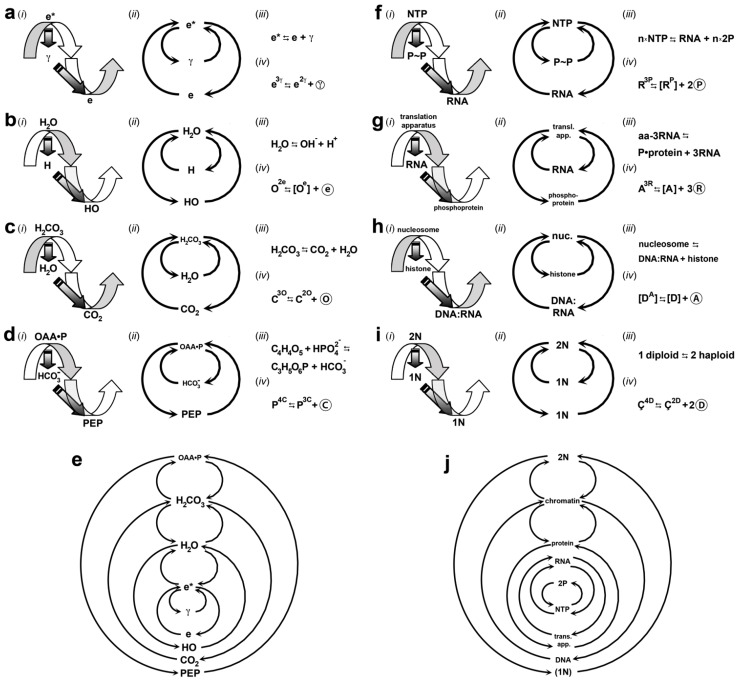
Understanding singularities. (**a-d, f-i**) Each singularity (gyre center) is represented as follows: (*i*) Gyrosystem; (*ii*) Matrioshkagyre; (*iii*) bidirectional, linear reaction or process; (*iv*) gyrequation. (**a**) Primary (1°) electrogyre(**b**) Alternoxygyre (**c**) Primary (1°) carbogyre (**d**) Alternaphosphogyre; n = any positive integer; P~P is pyrophosphate (**e**) Matrioshkagyre of the presented electro-, oxy-, carbo-, and phosphogyres (**f**) Secondary (2°) ribogyre (**g**) Tertiary (3°) aminogyre. Translation apparatus is the same as aa-3RNA (**h**) Alternagenogyre (**i**) Hapcellulogyre. Here, 1N and 2N represent chromosome content (**j**) *en face* Matrioshkagyre of the presented ribo-, amino-, geno-, and cellulogyres. Note how the Matrioshkagyre form reveals the nested thermodynamics and accurately positions one physical, chemical, biochemical, or biological process related to another. Acronyms, symbols, and models are defined in Table 1 and Table 2, Figure 1 and Figure 2.

To facilitate comprehension of the photon-as-singularity concept, I have presented it in four different ways in Figure 3a. The first fits the gyromodel to the evidence about the photon to the oscillating electron energy states (Figure 3a (*i*)), the second reveals the antichiral nature of and nesting of photons and electrons (Figure 3a (*ii*)), the third shows the known physical reaction (Figure 3a (*iii*)) and the fourth is the primary electrogyre as a gyrequation (Figure 3a (*iv*)). Looking ahead, I employ these four approaches for one exemplar of each other gyrosystem singularity.

**Figure 4:**
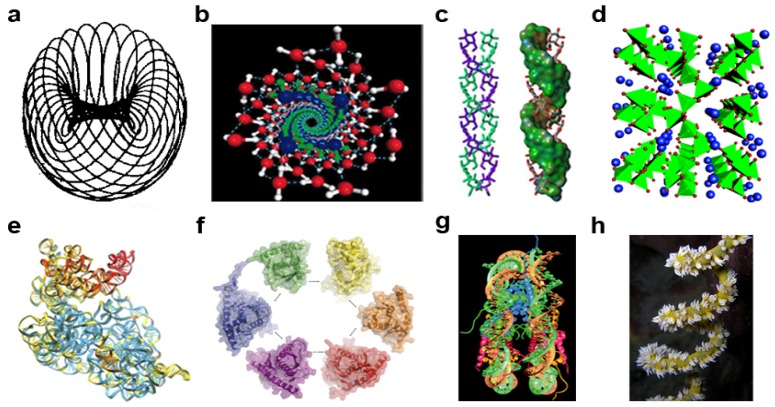
**Gyrosystem Forms.** (**a**) Electrogyre. Atomic chirality pictorially represented (oblique view) as electron probability current density for a hydrogenic 2*p*_1/2_ stationary state Reprinted and minimally adapted with permission from [212]. © 1998 American Association of Physics Teachers. (**b**) Oxygyre. A snapshot of quenched molecular coordinates of nano-ice. Reprinted from [213]. © 2006 by The National Academy of Sciences of the USA. (**c**) Carbogyre. Amylopectin, or glucose polymers with α(1→4) glycosidic bonds. Stick (left) and space-filling (right) models show how glucose polymers assemble into antiparallel helices. Reprinted from [214] with permission from Wiley. © 2010 WILEY-VCH Verlag GmbH & Co. KGaA, Weinheim. (**d**) Phosphogyre. Crystal structure of γ-Ca(PO_3_)_2_ showing unidirectional helical polyphosphate chains stacked in alternating perpendicular directions; Ca, blue; O, red; PO_4_ tetrahedra, green. Reprinted from [215] with permission from Wiley. © 2005, American Chemical Society. (**e**) Ribogyre. Composite structure of 16S rRNA compiled by comparing vacant *Escherichia coli* and tRNA-occupied *T. thermophilus* ribosomes. Note how the RNA right-handed double helices compactify into a matrix. Reprinted from [216] with permission from Elsevier. (**f**) Aminogyre. Crystal structure of the RNA exosome complex is a cyclical hexamer of α-helix dense RNase PH subunits. Reprinted and minimally adapted from [217] with permission from Elsevier. (**g**) Genogyre. Nucleosome architecture is a right-handed DNA double helix wrapping in a left-handed manner around a histone octamer. Reprinted by permission from Macmillan Publishers Ltd: *Nature* [218], © 1997. (**h**) Cellulogyre. Photograph of *Cirripathes spiralis*, a coral species. Image by N. Hobgood; licensed under the Creative Commons Attribution-Share Alike 3.0 Unported license.

Two gyraxioms help clarify the asymmetric and oscillating spin and chirality of leptons [72]. Specifically, 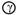 is predicted to be a dextragyre that imparts a dextral spin on **e** (G_XII_) whereas gyrosystemic balance occurs by the particle’s trajectory being levoral (G_VI_). Together, these gyraxioms and this gyrosystem (Figure 2a) explain the chirality of atoms ([73]; Figure 4a). Recall that each symbol in the gyrequation represents one or many particles and that each gyrosystem and -equation can be compressed or expanded accordingly. On this matter, the electrogyre accommodates the three generations of leptons [74], where the proximity to the gyradaptive singularity dictates the stability and energy of the particle.

*Higher-order Lepton Organization*. The gyrobase of the ***secondary electrogyre*** (Figure 2a (*ii*)) represents a low-energy electron monomer (unpaired electron), dimer (lone pair, Cooper pair), trimer, or polymer (***electronexus***) in any non-hydrogen atomic orbital [75,76]:

[e^γ^]_n_ = e^γ^, e^γ^e^γ^, e^γ^e^γ^e^γ^, and e^γ^e^γ^e^γ^e^γ^e^γ^e^γ^…

where a photon (γ) is the gyrolink and the electron is the gyromodule. This model thus boldly contrasts with the current notion that electrons reside as either unpaired or paired entities. The mobilization of the hydrogen electron (primary electrogyre) or any other sole electron to and from other electronexuses (secondary electrogyre) is modeled thusly:

e^2γ^ + 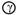 ⇆ e^3γ^ ⇆ [e^γ^] + 2
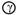


Compressing the gyrequation, I have

e^2γ^ + 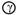 ⇆ [e^γ^] + 2
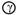

and balancing gives the new gyrequation,

e^2γ^ ⇆ [e^γ^] + 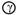


This 1°/2° schema illustrates the singularity (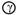) as metabolizing the electronexus. The further IEM is from the singularity, the lesser the attractorepulsive effect on it. In other words, the more distal IEM has a reduced electronexus cycle rate and forms longer, more stable electronexuses. This gyromodular organization thus explains the origin and emergence of atomic orbitals of all chemical elements and why there exist a greater number of electrons (2, 8, 18, 32…) in outer orbitals, or “shells [77].” The singularity, modeled dextrally to homeostatically balance the left-handed electrogyre, is predicted to induce electronexuses to form right-handed helices (G_XII_) called ***electrohelices***. Because an electrohelix in one atom exerts attractorepulsive effects (the electrohelix *is* a gyrating system) on free electrons and electrohelices in other atoms, this gyrosystem explains the emergence of inorganic chemicals that are necessary for the origin and evolution of life [78,79].

*Fermi Gas and Liquid States*. When visible energy is extracted from or unavailable for the electrogyre, the particles stop cycling between gyrosystates—this models a Fermi gas [80,81]. The Fermi liquid state, presently modeled mathematically (for instance, [82]) is modeled here as a three-dimensional crosslinked network of gyrobasal electrohelices—constantly undergoing metabolism, thus explaining fluctuating quantum “stripes” [83,84]—that are predicted to form a matrix, an ***electromatrix***. Fermi gases and liquids bear the signature of the electrogyre in their dynamic vortices [85,86,87,88,89].

*Electromagnetism*. Electromagnetism is a fundamental force of nature [90]. The primary electrogyre affords a new view of how this force emerges in the universe: repulsion of photons from and by the photonic singularity onto the electron depicts electricity,

e^2γ^ + 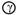 → e^3γ^
whereas photonic attraction into the singularity depicts magnetism,

e^3γ^ → e^2γ^ + 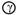


Thus, electro-magnetism can be rewritten as gyral repulsion-attraction [91]. Since the attractorepulsive force of any one electrogyre adapts to another *via* its singularity, the electrogyre models how changes to a magnetic field generate an electric field and *vice versa*. In response to incoming visible energy, the photonic singularity expands within a spatiotemporally restricted electrogyre. At a critical threshold, this expansion causes electrogyre collapse, accompanied by a quantized photon emission that thermodynamically flows and fractalizes through proximal electrogyres. Planetarily, this models a lightning strike and pre-lightning emissions [92,93,94]. Furthermore, given that the electrogyre expands omnidirectionally from within to without, it manifests as a high-energy, unstable electromagnetic sphere (***electrosphere***) that filled with light; this explains the enigmatic nature of ball lightning [95,96].

*Quantum Gravity*. The electrogyre unifies QM and GR. QG is modeled as the attractive force of the photonic singularity (a spacetime vortex itself (G_I_)) on the electron particle. The enigmatic wave-particle complementarity [97] of the electron is clarified because a gyrating particle oscillating between two states, creating the waveform (G_II_, G_V_). Moreover, the electrogyre (Figure 2a) shows how an experimentalist can examine quantal (particle) properties *or* the gyre (wave) properties, but not both at the same time. Given that the electron oscillates near the speed of light, the gyrostates of one ***electrocycle*** are difficult to detect but are predicted by theory. Electron observation requires photons. Examination induces visible energy exchange in the gyradaptor and, as long as photonic input is maintained, the particle arrests its gyratory motion (collapses its wavefunction) in the gyrapical state; this explains the measurement problem [98,99]. The Heisenbergian uncertainty of knowing two distinct particle characteristics simultaneously (e.g., position and trajectory [100]) is explained by the generic gyromodel itself (Figure 1b; 2.4.2.) and by the innate adaptation (electrogyre learning is ***electrognosis***; electrogyre memory is ***electromnemesis***) of the gyrosystem in response to interrogation. Given that the electrogyre is a theoretical solution to QG, it affords a radical perspective on the core elements of reality and on the primal role of gravity in the evolution of life [101,102].

*Planetary core*. The current idea for how planets originate in the cosmos, *in medias res*, is through the cooling of an interstellar gas cloud followed by the gravitational accretion of particles into larger and larger aggregates. As the story goes, the gravitational sink of aggregates leads to accelerative accretion and the emergence of protoplanets [103]. High-pressure and temperature experiments, seismology, and fluid dynamics modeling have led scientists to infer properties of Earth’s core [104] and to make statements about the origin of Earth in particular [105]. However, perhaps apocryphally, Einstein considered geomagnetism one of the most important unsolved problems of physics, implicitly calling the accepted model into question.

Using the gyromodel, the inner core of a planet is modeled as a ***macroelectrogyre***, such that, for example,

(e^3γ^ ⇆ e^2γ^ + 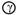)_n_ and (e^3γ^ ⇆ [e^γ^] + 2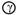)_n_
where 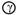 models a ***macrophoton***, and n is an inordinately large number of components in the gyrosystem. This gyrequation reveals that the planetary core (***macroelectron***) emerges from a macrophoton in a fashion similar to leptonic emergence from a photon. As the macroelectrogyre has the vectorial, spinning, flowing form, it is compatible with the geophysical evidence regarding precession [106]—where a planet rotates on its own axis just like a spinning gyroscope [107]—and the geomagnetic field [108], which is modeled cosmically as it is atomically (Figure 4a). Further, because the excited state of the macroelectrogyre is shared by all the majorgyres, the secondary electrogyre-derived ***macroelectronexus*** emerges within and models the inner core of a planet. Being an adaptive supermassive helix, the macroelectronexus is consistent with the notion of a geodynamo in Earth’s core [109] yet diverges from the current idea that the core is liquid iron [110]. Finally, the gyrobase of the ***tertiary electrogyre*** (Figure 2a (*iii*)) models a planetary core, [e], that loses its macrophotonic support. The thermodynamic switch between the two most extreme majorgyrostates (e^3γ^ and e) parsimoniously models geomagnetic reversal, a periodic geophysical event that has not been observed and thus has engendered much speculation [111,112,113].

*Planetary orbit*. As the macroelectrogyre (planet) spins on its own axis as a consequence of the thermodynamic flow from its internal macrophoton, it rotates in an observable higher-order gyre (***ohiogyre***) around a central, capacious, more exergic macrophoton (star) from which it emerged. The ohiogyre provides a unique perspective on how energy emitted by a star influences the evolution of a planet: macrophoton expulsion (solar wind [114,115,116], coronal mass ejections [117,118]) repels the macroelectrogyre into a high energy state known in celestial mechanics as perihelion. The macroelectrogyre adapts by mobilizing, metabolizing, storing, and changing the energy within itself and expanding. Being a dissipative system, the macroelectrogyre also disperses some of the energy as heat into space, thereby falling to a relativistically lower energy state known as aphelion. Thus, as modeled by the ohiogyre, quantized macrophoton influx induces macroelectrogyre oscillation between excited and ground states, explaining both the periodicity of planetary orbit and why a planet does not gravitationally collapse into a star. Finally, as with atomic orbitals, in planetary orbits, the attractorepulsive effects diminish the further away from the macrophoton singularity. The macroelectrogyre predicts that increased size and slower orbit of distal planets relative to proximal ones (as in the Solar System) corresponds to the composition, length, and stability of macroelectronexuses.

*Antimatter*. An outstanding question in physics is why there is so little antimatter in the physical universe [119,120]. Microcosmically, the tertiary electrogyre (Figure 2a (*iii*)) shows the electron cycling out the thermodynamic support of the triphoton. Given synchiral organization of the tertiary majorgyre gyrobase (G_VII_, G_XII–2_), the electron destabilizes and ultimately collapses due to the synchiral sub_2_gyre (not shown) in lieu of the antichiral subgyre, modeling the positron. The extreme creatodestructive swing of the electrogyre thus provides an explanation for the fleeting presence, or absence, of antimatter in the universe.

*From Visible Matter to Water.* The electrogyre models, explains, positions, and predicts fundamental physical phenomena and provides a framework for the origin and evolution of the Solar System in the Milky Way Galaxy [121]. Although I intentionally focused on data acquired from experiments and observations of the Solar System, the electrogyre is flexible enough to be tested against evidence regarding any star and planetary system in the universe [122]. Compressing the primary, secondary, and tertiary electrogyres reveals how leptons and photons interconvert:

e^2γ^ ⇆ [e^γ^] + 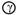 ⇆ [e] + 2
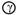


Importantly, this empirically established and symbolically represented relationship precisely conforms to the first law of thermodynamics, known as the physical law of the conservation of energy [123].

The universe has been expanding since its origin and universal expansion is currently accelerating [124]. I explain this expansion (*i.e*., cosmological inflation [125]) as the omnidirectional repulsive force of the photon on the electrogyre and, consequentially, as the repulsive force of the electrogyre on all of the supervenient forms of matter and information in the evolving universe. (I qualify my explanation by noting that this framework does not incorporate or explain dark energy, dark matter, and sub-atomic particles [126,127,128].) This theory predicts that, during expansion of the universe, the electrogyre—an ordered mélange of elements and inorganic chemicals—achieved a thermodynamically unstable state of high energetic potential, whereupon, the electrogyre collapsed. This disruption and release of kinetic energy, in light of continued expansion, predicates a significant, far-reaching change in universal evolution: the emergence of water.

### 3.2. Water

The emergence of novel IEM forms is a grand evolutionary and philosophical problem [129,130]. Theoretically solving this problem *should*, in principle, be possible, since evolutionary events are constrained by natural laws, physical forces, and chemical elements that lead up to them. Yet biological macroevolution is thought to work quantally, “explosively [131].” At this juncture, I require an answer to the following question: What fundamental feature of life evolves following and from visible, inorganic matter?

As water is the single largest component (70–90%) of the living cell, the fittest answer is water [132]. Known as “the universal solvent,” water is one of the simplest chemical molecules, consisting of oxygen (O) and hydrogen (H). In spite of its chemical simplicity, its complexity is legendary: a brutally honest, erstwhile *Nature* editor opined, “no one understands water [133].” In this subsection, I squarely face the emergence of water with a reified systems model called the ***oxygyre***. The oxygyre not only models the origin and nature of water in the universe and in the living cell but also fits data related to celestial oxides, oceans, and moons.

I make two crucial points prior to proceeding. First, in modeling the chemistry and thermodynamics of water, the reader must view the hydrogen atom from the standpoint of its sole electron (denoted here as the quantized particle ⓔ; Figure 2a (*iv*)) rather than its proton (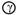). Second, given theoretical expansiveness and particle-quantum relations (G_III_), **e** has alternate applications beyond the hydrogen electron; these are discussed later in this subsection.

*Origin of water*. There have been many clues and ideas regarding the origin of water on Earth and in the universe [134,135,136], but no solution. Modeling the origin of water here requires a brief reminder of electrogyre characteristics (3.1). As the singularities of the primary and secondary electrogyre are uniphoton (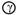) and diphoton (2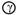), respectively (Figure 2a (*i*) and (*ii*)), the singularity of the secondary electrogyre exerts a greater attractive force on the triquantal excited state electron (e^3γ^) than does that of the primary electrogyre (recall that majorgyres share the triquantal state). Thus, the electrophilicity of the oxygen atom, like all other electron sinks, is modeled by the relativistically higher magnetism of the secondary electrogyre. Moreover, as gyromodeled, the relative proximity of an electron or electronexus to the singularity affects its metabolic rate.

The gyromodel dictates that the ***oxyon*** (the quantum/particle form of the oxygyre, Figure 2b (*iv*)) particle spins levorally yet gyrates dextrally to offset electrogyre chirality (G_VI_, G_VII_)—thereby diminishing the rate of gyrostatic oscillations and homeostatically balancing the gyrosystem and the universe. In other words, the chemical molecule known as water emerges quantally from *within* the pool of elements during the aforementioned electrogyre collapse. Gyromodelling water would thereby be axiomatically compatible: a focagyre (oxygyre) is dependent upon a subgyre (electrogyre) and is an emergent IEM form (G_VIII_, G_IX_).

*Phase transitions*. Where chemical molecules are concerned, there are three main phases, or distinct states of matter, which have essentially uniform physical properties throughout: solid, gas, and liquid. The current approach for describing phase transitions—thermodynamic transformation from one state of matter to another—is mathematical [137]. Despite much progress, the math is incapable of explaining *why* there are three main phases and *precisely* how they interconvert. Here, I fit evidence about phase transitions of water to the majorgyre frame.

Microcosmically, the ***primary oxygyre*** (Figure 2b (*i*)) models the molecular oscillation between H_3_O (O^3e^; gyrapex) and H_2_O (O^2e^; gyrobase). Unfortunately, confusing matters slightly, this is written in chemical notation as

H_3_O^+^ + e^−^ ⇆ H_2_O + H

because the trielectron oxygen (H_3_O) is a challenge to detect due to its instability (H_3_O^+^ + e^−^). This gyrosystem parsimoniously depicts sublimation (transformation from solid to gas) as attraction by the singularity, pulling the electron off the oxygen atom and reconstructing the hydrogen atom (ⓔ; gyradaptor):

O^3e^ → O^2e^ + ⓔ

Deposition (transformation from gas to solid) is modeled as repulsion of the electron onto oxygen:

O^2e^ + ⓔ → O^3e^

Thus, O^3e^ represents ice and O^2e^ is water vapor. The primary oxygyre models these phase changes in living systems [138] and, as it is a general theory, on a macrocosmic scale in glaciers, hail, sleet, snow, clouds, and fog in both early evolution of planets and present-day atmospheres [139,140], *i.e*.:

(O^3e^ ⇆ O^2e^ + ⓔ)_n_

Understanding the other phase and phase transitions of water requires fitting data onto the secondary majorgyre. The gyrobase of the ***secondary oxygyre*** (Figure 2b (*ii*)) models an oxy-electron monomer ([O^e^] fits OH, or hydroxyl ion), dimer (fits H_2_O_2_, hydrogen peroxide), and any length of polymer, an ***oxynexus***, shown as:

[O^e^] = O^e^, O^e^O^e^, O^e^O^e^O^e^O^e^O^e^O^e^ …

As the gyromodel stipulates, oxynexuses are continually being catabolized (created) and anabolized (destroyed) by the electron singularity. Given G_XII_, the oxynexus is predicted to assemble into a left-handed ***oxyhelix***. What do oxynexuses and oxyhelices model? These structures represent how the liquid phase of water is organized [141,142]. Large-scale ordering of these oxynexuses are matrices, called ***oxymatrices***; on a planetary scale, a ***macroxymatrix*** is the theoretical term for an ocean. Given the theoretical finding that macroxymatrices are a direct evolutionary and thermodynamic consequence of the macrophoton (Sun) and macroelectron (Earth), this helps unravel the faint young sun paradox [143,144]. The secondary oxygyre models melting (solid to liquid) as gyradaptive attraction by the dielectron:

O^3e^ → [O^e^] + 2ⓔ

Freezing (liquid to solid), in contrast, is gyradaptive repulsion by the dielectron:

[O^e^] + 2ⓔ → O^3e^

Combining the primary and secondary oxygyre in a gyrequation gives

O^2e^ + ⓔ ⇆ O^3e^ ⇆ [O^e^] + 2ⓔ

thus tidily modeling the interconnectivity of the three phases of water in the biogeochemical water cycle,

H_2_O + H ⇆ H_3_O ⇆ OH + H_2_

termed here a ***macroxygyre***: solid (O^3e^), gas (O^2e^), and liquid ([O^e^]). One full cycle of any oxygyre is called an ***oxycycle***. Therefore, this model explains the biogeochemistry of water ([145], and see below) and is a heuristic for framing phase transitions for any other forms of visible matter. Please note the modeling of hydrogen gas (where 2ⓔ = H_2_), an important molecule in chemosynthesis and planetary organization and formation [146,147,148,149]. Compressing the gyrequation, I have

O^2e^ + ⓔ ⇆ [O^e^] + 2ⓔ

and balancing reveals

O^2e^ ⇆ [O^e^] + ⓔ

providing a theoretical schema for the gas-liquid phase transition. I use this specific transition to illustrate the electron-as-singularity concept (Figure 3b). Rounding out the phase transitions of water, condensation (gas to liquid) is modeled as electron attraction from the oxygen electronexus (O^2e^) into the singularity (ⓔ), forming liquid water ([O^e^]):

O^2e^ → [O^e^] + ⓔ

Vaporization (liquid to gas) is modeled as particle repulsion from the singularity to the oxygen electronexus, orbiting the atomic nucleus:

[O^e^] + ⓔ → O^2e^

Reiterating, as conjunctively modeled by the oxygyre, attractorepulsive electron flow through the O orbitals is liquid (fluid water); electron capture by O is solid (ice); electron eviction by O is gas (water vapor).

*Nature of water*. I now discuss a few of water’s enigmatic characteristics in light of the model. First, as the oxygyre expands and contracts omnidirectionally in response to an influx of visible energy within the electron singularity (Figure 2b (*iv*)), the oxygyre concomitantly fills out the hyperbolic geometry of a sphere (***oxysphere***). The binary attractorepulsive effects—photon-electron, electron-oxyon, and photon-oxyon—explain not only the uniform but adaptive spherical shape of a water droplet but also the general surface tension of water [150,151]. Second, because a gyrating oxyon particle assembles into higher-order quantized structures that also gyrate and oscillate (G_II_, G_III–1_, and G_XII_), this provides a theoretical basis for the tempo and mode of the water oscillatory waveform [152,153]. Third, the controversial if scientifically heretical concept that water has memory [154,155] is supported by theory, as the oxygyre undergoes ***oxygnosis*** and ***oxymnemesis*** as part of its adaptational cycle (2.4.4.). Fourth, Matrioshkagyre relationships of the photon, electron, and the oxygyre demonstrate how visible energy is stored within water and explain the high heat capacity of water—an alternative view than that given by mathematical models [156]. Fifth, the hemispherically antichiral oceanic gyres in the Atlantic and Pacific oceans [157,158] are modeled as a consequence of the attractorepulsive forces exerted by the vectorial macroelectronexus at the core of the Earth on the macroxymatrix. Sixth, the vortical structure seen throughout the oceanic and atmospheric world (e.g., hurricanes, maelstroms, tornados) is modeled by the oxygyre and is even detectable in nano-ice (Figure 4b; [159]).

*Oxygen species*. The origin of oxygen in early Earth’s atmosphere is thought to be a solved problem: cyanobacteria emerged in an anoxic environment to produce molecular oxygen (O_2_) as a byproduct of photosynthesis [160,161]. However, since there is no way of performing experiments on early Earth, this idea cannot be *directly* validated or refuted. Departing from this provisional solution, I applied the oxygyre to the problem. The ***tertiary oxygyre*** (Figure 2b (*iii*)) depicts trielectron cycling from, to, and through the singularity, revealing how the oxygen species—elemental oxygen and reactive oxygen radical, O or [O]_1_, molecular oxygen, OO or [O]_2_, and ozone, OOO or [O]_3_—originate and oscillate to and from the solid phase of water (O^3e^):

O^3e^ ⇆ [O] + 3ⓔ

Note that, the photon is the gyrolink between the oxygen gyromodules (G_XI_), thus providing insight into how the oxygen-dense atmosphere adapts to the Sun’s efflux [162]. Moreover, the tertiary oxygyre dictates an early emergence of oxygen species on Earth and their necessity to the evolutionary origin of life [163,164]. Modeling the movement of water vapor through living systems and the atmosphere in the oxygen cycle (another macroxygyre) requires combining the primary and tertiary oxygyres,

O^2e^ + ⓔ ⇆ O^3e^ ⇆ [O] + 3ⓔ

which compresses and balances to

O^2e^ ⇆ [O] + 2ⓔ

uncovering the 1°/3° alternagyre. Modeling of the relationship of liquid water and oxygen species is modeled by compressing secondary and tertiary oxygyres:

[O^e^] + 2ⓔ ⇆ O^3e^ ⇆ [O] + 3ⓔ

which reducesand balances to

[O^e^] ⇆ [O] + ⓔ

revealing the 2°/3° alternagyre. Finally, given that the tertiary oxygyre oscillates between extreme gyrostates (O^3e^ and [O]) over a geological time scale, this explains not only why and how Earth experienced several intervals of intense glaciation (O^3e^) known as “snowball Earth [165,166]” but also the appearance of free oxygen ([O]_2_) in Earth’s atmosphere, like the “great oxidation event [167].”

*Oxide Geochemistry and Geophysics*. The necessity of modern geochemical cycles to bio-organismal existence on Earth conceals a vital thermodynamic connection between planetary and cellular evolution [168]. Still, it is inappropriate to harbor any assumptions related to such connections but allow theory to inform them. I reported in 3.1 that the macroelectron, the planetary inner core, harbors within itself the potential for all chemical elements and inorganics. I now frame a second major leap in planetary evolution: the formation of the outer core, mantle, and crust.

The Earth’s mantle and crust are highly enriched in oxides, all of which are accounted for by the primary and secondary oxygyre. For example, in the primary oxygyre, O^2e^—where e here represents the secondary electrogyre and hence any chemical elements (G_III_)—models the low abundance compounds Na_2_O and K_2_O (each ion represents 1e and thus two make the compound); it also models CaO, FeO, and NaO (each ion represents 2e), which are ~3, 7.5 and 48% of the Earth’s mantle and ~6, 7, and 5% of the crust, respectively [169]. The secondary oxygyre also models the major makeup of Earth’s outer core [170], mantle, and crust. As the outer core has been proposed to be liquid [171], a macroxygyre models its thermodynamically fluid and dynamic character. [O^e^]_2_ models SiO_2_, this being ~46% of the mantle and ~61% of the crust and [O^e^]_3_ accounts for Al_2_O_3_—~4% of the mantle and ~16% of the crust [169]. Supporting a more broad application of the oxygyre to understanding crustal organization and dynamics, oxygen is ~50% of crustal mass and the crust itself is > 99% oxides [172].

Geophysical theories have been quite successful, but anomalies and inexplicable phenomena have hinted at their limitations [173]. I submit that there are several noteworthy geophysical features that can be deduced from the nesting of the macrophoton within the macroelectron within the macroxyon. First, in response to photon influx from solar emissions, the macrophoton swells within the macroelectron. As photons step-decelerate to leptons (3.1), the macroelectron, in turn, organizes, stores, and emits IEM from within to without. Macroelectron expansion elicits macroxyogyre expansion to accommodate the IEM influx. This theoretical scheme prescribes that the Earth—and, as predicted by theory, all planets and planetary bodies—formed by expansion as opposed to accretion [174]. This prescription conforms with the expanding Earth concept [175] and thus addresses numerous problematical issues in the fields of volcanism [176,177], landmass formation [178,179,180], continental drift [181], and seismology [182,183]. On the most latter point, I elaborate on how the Matrioshkaquantal structure of the Earth relates to seismic activity. During expansion, the macrophoton singularity (within the macroelectron core) reaches a local thermodynamically unstable state, detected as pre-earthquake signals [184], whereupon it re-equilibrates. A quantal emission of energy ripples outward, moving as focused solitons [185] through the macroelectron and macroxyon layers, *i.e.*, spherical shells. Following passage through these gyrosystems on macrocosmic and microcosmic scales (all of the unique chemical elements and molecules throughout the inner and outer core, mantle, and crust), the quantum of energy arrives at a spacetime point of criticality. The unpredictability, fractalization, and rippling solitonic flow of gyrosystems resolves the enigmatic characteristics of earthquakes [186,187]. Here stands a formal theoretical relationship between solar emissions and seismic activity, confirming a long-standing idea [188].

*Lunar Formation*. The favored hypothesis for the formation of Earth’s Moon is from planetesimal impact on a proto-Earth proceeded by matter ejection, accretion, and gravitational capture [189,190]. However, the question of lunar origin has not been settled since there are competing, albeit antiquated hypotheses [191,192]. I also discovered the stunning admission that, “…shamefacedly, [astronomers] have little idea as to where [the Moon] came from. This is particularly embarrassing… [193].” The oxygyre models the Moon as a macroxyon that has a macroelectron within itself; this simple gyrosystem accounts for the known chemical composition of the Moon surface, oxides [194]. Regarding lunar origin, the macroxyon that *is* the Moon emerges from the macroelectron that *is* the Earth, concomitant with the emergence of Earth’s macroxyon [195,196].

Several additional points can be derived from this gyrosystem. First, the oxygyre explains water on and in the Moon [197,198,199]. Second, the gyrating effects of the macroxygyre model the rotation of the Moon on its axis. Third, the path of a less exergic macroxyon (Moon) around more exergic one (Earth) follows an ohiogyre path, or lunar orbit. Fourth, this oxygyre provides insight into how tidal cycling is linked to lunar orbit and axial rotation [200] since the Earth’s oceans (macroxymatrix) and Moon itself (a macroxyon) exert complementary attractorepulsive forces. Fifth, this theoretical union also helps clarify short-term chronobiological ([201]; see 3.8) and long-term geophysical [202] relationships. Sixth, the craters that cover planetary, lunar, and satellite surfaces [203,204,205]—most if not all of which are near-perfect circles—bear the signature of the macroelectron singularity and its strong thermodynamic force on the oxygyre [206].

*From Phased Matter to Organic Matter.* In this subsection, I restricted my attention to Earth for a very important reason: life as I know it evolved on Earth and thus Earthly life is what I model. This rationale guides fact fitting in subsequent subsections. Still, the oxygyre is a cohesive and consistent model for the emergence and cycling of oxygen species in the early evolution of the Earth [207,208], different from any computer model [209]. The oxygyre is predicted to be fruitful vis-à-vis modeling planets and moons of the Solar System and Earth-like planets and star systems throughout the cosmos [210,211].

The positioning of information, energy, and matter in the oxygyre relative to the electrogyre accounts for two sets of phenomena originally thought to be modeled independently: *modern* (present-day) celestial mechanics, geophysical processes, and cellular thermodynamics of water and *remote* (evolutionary) planetary growth, lunar emergence, and the origins of water and molecular oxygen that are necessary for life’s origin. The next gyrosystem must emerge from within the electrogyre and oxygyre; that is, supragyre emergence and thermodynamic metabolism are dependent upon the oxygyre (G_VIII–1_). On this axiomatic constraint, I fit water and oxide cycling through organic biochemicals and geochemical systems as the next emergence.

### 3.3. Organic Matter

All life that has been identified by the scientific method is carbon-based. In the absence of a consensus explanation for the necessity of organic matter to life, other hypothetical types of biochemistry not reliant on carbon have been postulated (especially silicon, [219]); and yet, none have been identified. Any theory of life, to be considered meritorious, would be expected to provide an explanation for *why* life is carbon-based and shed light on whether or not alternative biochemistries are possible [220].

Here, I gyromodel evidence regarding carbon compounds on Earth and in the cosmos and regarding organic biochemical pathways in the extant cell. The ***carbogyre*** is the gyrosystem that emerges from within the electrogyre and through the oxygyre. In viewing the carbogyre (Figure 2c), the reader should be aware that only two IEM species are permitted in the symbolic architecture of a gyromodel. That is, the carbogyre displays carbon moieties (C), oxygen moieties (particle, O, and quantum, Ⓞ) but does not display electrons (e and ⓔ) or photons (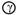)—even though they are present—for gyromodel consistency and clarity.

*Emergence and Cycling of CO_2_*. The origin of carbon dioxide (CO_2_) in Earth’s atmosphere is a matter of much speculation [221,222]. Understanding its origin is of great scientific import, as plants literally construct themselves from, and maintain themselves with, CO_2_ [223,224]. The *primary carbogyre* (Figure 2c (*i*)), written in the gyrequation

C^3O^ ⇆ C^2O^ + Ⓞ

exquisitely fits the chemical reaction

CH_2_O_3_ ⇆ CO_2_ + H_2_O

which is interconversion of carbonic acid with carbon dioxide and water vapor—relevant both astrophysically and terrestrially [225]. Here, C^3O^ is the gyrapex for all majorgyre, C^2O^ is the gyrobase, and Ⓞ is the gyradaptor. Modeling water as the singularity is presented four different ways in Figure 3c. Like the aforementioned gyrapices (triphoton, trielectron), the trioxygenated carbonic acid is an extremely high energy, thermodynamically unstable compound [226]. The cycling of carbon dioxide in the early and present day Earth atmosphere and biosphere is thus vectorially modeled as the attractive force of the oxyon singularity on the mIEM oxyon particle (dehydration reaction):

C^3O^ → C^2O^ + Ⓞ

formation of carbonic acid is due to the repulsive force of the oxyon (hydration reaction):

C^2O^ + Ⓞ → C^3O^

This schema shows that, even though the electrogyre accounts for the interactions between and among the electrons in all elements, the primary carbogyre is a dedicated gyrosystem for modeling interactions between and among carbon and oxygen atoms/compounds. A clearer picture of how water is the singularity of the primary carbogyre can be found in Figure 3c. An explanation of the origin of CO_2_ requires the introduction of additional carbogyrosystems and is discussed below.

*Atmospheric CO_2_ Levels*. Keeling was the first to precisely measure monthly atmospheric CO_2_ levels on Earth from the middle of the 20^th^ century onward, leading to production of his eponymous curve [227]. Two features of this curve are noteworthy: CO_2_ levels are both increasing and oscillating. While the increase in CO_2_ has been argued to be a hallmark of global climate change from burning fuel [228,229], the oscillating levels are thought to reflect the natural CO_2_ flux into and out of the oceans and biosphere. The carbogyre explains both phenomena simultaneously: *macrocarbogyre* expansocontraction driven by macroxyon expansocontraction and attractorepulsion models the increasing (expansion) and oscillating (spacetime carbogyration) levels of atmospheric CO_2_. Vital gas exchange in life can now be modeled by nesting the oxygyre and carbogyre in a gyrequation. Given G_I_,

Ⓞ = (O^2e^ ⇆ [O] + 2ⓔ)

The oxyon can be nested in the primary carbogyre thusly:

C^3O^ ⇆ C^2O^+ (O^2e^ ⇆ [O] + 2ⓔ)

which models the attractorepulsive relationship between O_2_ and CO_2_ (both in bold) found in many living systems:

2H_2_CO_3_ ⇆ 2**CO_2_** + (2H_2_O ⇆ **O_2_** + 2H_2_)

This theoretical formula indicates an early evolutionary emergence for respiratory gas exchange that exists in humans, animals, and plants; this solution is well beyond other ideas [230,231]. A variation on this nested arrangement of these two gyrosystems is found in Figure 3e.

*Acid-base homeostasis*. The proper balance of acids and bases is necessary in cells, in blood [232], and in the Earth’s oceans [233]. Yet, there is no standard model for how acid-base homeostasis evolved. By adding H^+^, the hydron and HCO_3_^−^, bicarbonate, to the reaction above, I get:

H^+^ + HCO_3_^−^ ⇆ H_2_CO_3_ ⇆ CO_2_ + H_2_O

From this known chemical reaction, I now model the thermodynamic relationships of the photon, electron, oxyon, and ***carbyon*** (quantized particle; Figure 2c (*iv*)), written out to accommodate all of gyrosystems in the fractalized gyrequation:

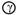
 + C^3O^2e^1γ^^^ ⇆ C^2O^2e^2γ^^^ ⇆ C^2O^ + O^2e^2γ^^

Thus, this theoretical framework neatly dispatches and unifies the microcosmic and macrocosmic origin of acid-base homeostasis.

*Carbohydrates*. One chemosynthetic step needed for the origin of the living cell is carbohydrate production [234,235]. While it is true that photosynthesis generates a prominent carbohydrate, glucose, several features of this photochemical process have yet to be theoretically clarified [236,237]. Given the structural constraints of the secondary majorgyre, in the ***secondary carbogyre*** (Figure 2c (*ii*)), [C^O^] is the gyrobasal IEM unit with potential to polymerize. Note that the oxyon is the gyrolink in the carbonexus (G_X_), but is, in fact, orbiting carbon atoms that are gyromodules. Remember that

[C^O^] = C^O^, C^O^C^O^, C^O^C^O^C^O^ …

such that [C^O^]_1_ is CH_2_O—formaldehyde, the most volatile, ubiquitous, and simplest aldehyde that is the unit component of organic polymers [238,239,240]. [C^O^]_2_ is C_2_H_4_O_2_ (glycoaldehyde, an important prebiotic chemical [241]); [C^O^]_3_ is C_3_H_6_O_3_ (trioses, e.g., glyceradehyde, which has been suggested to impart chirality to biomolecules [242]; also models pyruvic acid, a hydrothermally reactive compound [243] and the energy source for the citric acid cycle under oxygenating conditions [244]); [C^O^]_5_ is C_5_H_10_O_5_ (pentoses, e.g., ribose, the nucleotide sugar [245,246]); and [C^O^]_6_ is C_6_H_12_O_6_ (hexoses, e.g., glucose and galactose, both notable biomolecules [247,248]). The secondary carbogyre models dehydration of any carbonexus as dioxyon attraction:

C^3O^ → [C^O^] + 2Ⓞ

hydration is modeled as dioxyon repulsion:

[C^O^] + 2Ⓞ → C^3O^

This ordering of ***carbonexus***es—the theoretical neologism for carbohydrates—is consistent with ideas that formaldehyde and glycoaldehyde are the starting points for carbohydrate metabolism [249]. Further validating the theoretical positioning of formaldehyde, compressing (but not balancing) the primary and secondary carbogyres, I have

C^2O^ + Ⓞ ⇆ [C^O^]+ 2Ⓞ

which, given the representational variability of the quantum, the left and right side Ⓞs model the primary and tertiary oxygyre, respectively, exactly fits the following well known reaction in the long-term carbon cycle:

CO_2_ + H_2_O ⇆ CH_2_O + O_2_ [250]

*Photosynthesis*. Photosynthesis is fundamental not only for plant life, but for all life on Earth. The current ideas about the origin and evolution of photosynthesis come from a mosaic of data from biochemistry, biophysics, bioinformatics and physiology [251,252,253,254], but these have not coalesced into a general theory. Multimerizing (n = 6) the prior gyrequation, I have,

(CO_2_ + H_2_O ⇆ CH_2_O + O_2_)_6_, or

6CO_2_ + 6H_2_O ⇆ C_6_H_12_O_6_ + 6O_2_

the photosynthesis reaction. The nesting of the photon within the electrogyre, and the electron within the oxygyre, and the oxyon within the carbogyre thus reveals a new algorithm for photosynthetic energy transfer.

*Organic cycles*. There are several organic cycles throughout the natural world whose origins have remained a mystery. These are now economically fit onto the carbogyre frame. On a cellular scale, the core details of several fundamental processes are modeled as oscillating carbogyres with varying carbon number and form (singularity excluded from the following bidirectional equations):

Glycolysis: C6 ⇆ C3 [255,256]

Citric acid cycle: C6 ⇆ C4 [257,258]

Calvin cycle: C5 ⇆ C3 [259]

This theory is thus consistent with prior ideas of the citric acid cycle being self-organizing [260]. On a planetary scale, the biogeochemical carbon cycle—the cycling of carbon through the air, oceans, soil, organisms, and sediments [261]—is a ***macrocarbogyre***. One complete cycle for an individual carbyon in this gyrosystem is a ***carbocycle***.

*Sugar Homochirality*. In 1860, Pasteur proposed an explanation for why sugars are asymmetric (D, right-handed) in living systems, called “chirality” by Lord Kelvin [262]. Despite many ideas for the origin of chemical chirality, there has been no satisfactory explanation. This theoretical framework shows that carbonexuses are only the D enantiomer because the oxygyre is dextral (Figure 2b and Table 2) and, based upon G_XII_, the subgyre exerts the formative, directional, vortical force on the matter in primary and secondary majorgyres of the focagyre. Notably, this model is consistent with the empirical observation that vortices induce chiral selection [263].

*Higher-order Carbohydrate Structures*. Carbonexuses have potential to form higher-order structures such as multimers (e.g., sucrose: C_12_H_22_O_11_), ramified polymers as in oligosaccharides and polysaccharides (e.g., starch, glycogen, cellulose, and chitin) and also empirically defined helices and matrices theoretically termed ***carbohelices*** (Figure 4c; [264,265,266]—predicted to be left-handed in living systems because sugars are right-handed (G_XII–3_)—and ***carbomatrices***, respectively. As starch complexifies, it oscillates between helical and latticed states [214]. Because both simple and complex carbohydrates are modeled by the adaptive and responsive carbogyre, this explains the mysterious property of the “feeling” of recognition saccharides [267].

*Hydrocarbons.* There are two competing theories to explain the origin of hydrocarbons on Earth and its relationship to life. The first, the mainstream biogenic theory, posits that hydrocarbons emerge as a natural result of cellular decomposition and biodegradation of buried organic matter [268]. Given the forcefulness with which this theory has been promoted, subterranean hydrocarbons and petroleum products are called by the moniker “fossil fuels.” The second, called abiogenic theory, suggests that organic matter emerges deep within the Earth, partially or largely independent of biodegradation [269,270,271]. Despite much inquisition [272], there has not been a satisfactory resolution of the biotic/abiotic debate. Can the carbogyre arbitrate this scholarly dispute?

In the ***tertiary carbogyre*** (Figure 2c (*iii*)), the trioxyon (3Ⓞ; gyradaptor) cycles on and off the carbon atom. In the absence of thermodynamic support of the oxyon, the gyrobase carbon atom establishes thermodynamic relationships the electrogyre (G_XI_). Hence, in one theoretical incarnation, the gyrobase of the tertiary carbogyre models hydrocarbons as carbonexuses where

[C]_n_ = C^e^, C^e^C^e^, C^e^C^e^C^e^, and C^e^C^e^C^e^C^e^C^e^C^e^….

[C]_1_ is CH_4_ (methane), a crucial and volatile natural gas that has biotic and abiotic importance [273,274]. Other important hydrocarbons such as ethane, ([C]_2_ is C_2_H_6_), propane ([C]_3_ is C_3_H_8_), butane ([C]_4_ is C_4_H_10_), and so on [275] fit here.

The tertiary carbogyre permits thermodynamic relationships with all chemical elements [276,277,278]. Some of these organic chemicals are biometabolized by a variety of microorganisms [279,280] and many if not most are found in petroleum [281]. The attractive force of trioxyon models oil formation during planetary evolution:

C^3O^ → [C] + 3Ⓞ

the countervailing repulsive force models its breakdown:

[C] + 3Ⓞ → C^3O^

The validity of the carbogyre is further supported by equating the primary and tertiary carbogyres:

C^2O^ + Ⓞ ⇆ [C] + 3Ⓞ

Compressing and balancing gives

C^2O^ ⇆ [C] + 2Ⓞ

which, in acknowledging that H_2_O (Ⓞ) was removed from the left-side of the gyrequation during compression, is consistent with the established, if overly simplified relationship:

CO_2_ ⇆ hydrocarbon + O_2_

a chemical reaction commonly found throughout the climatological, environmental, and energy literature [282,283,284]. Taken together, then, a macrocarbogyre models the deposition and metabolism of geophysical petroleum in the mantle and crust. As positioned here, the origin of hydrocarbons occurs spatiotemporally prior to the emergence of the living cell.

*Immiscibility*. The tertiary carbogyre provides an explanation for immiscibility—in this case the inhomogeneity of water and oil [285]—a necessity for the compartmentalized cell to exist. As revealed by the tertiary carbogyre, hydrocarbons harbor no oxyon. Rather, the trioxyon is found exclusively in the singularity, where it exerts a powerful repulsive force to collapse the gyrosystem from its most unstable, high energy state to its relativistically most stable, low energy state. The hydrophobic (water-fearing) relationship between and oil droplet (***carbosphere***) and water (oxyon) is thus due to this modeled repulsion. From this relationship I have an evolutionary consistent frame for modeling the origin of simple emulsions and non-phosphate membranes [286].

*Alcohol*. There is no extant general theory for the evolutionary origin of alcohols, although there are predictions regarding chemical constraints for their origin [287]. Here, modeling alcohols requires compression of the secondary and tertiary carbogyres, ultimately revealing the following gyrequation:

[C^O^] ⇆ [C] + Ⓞ

Given the inverse particle-quantum relationship (G_III_, G_III–1_), the oxyon in the unit [C^O^] can represent any primary, secondary, or tertiary oxygyre or combination thereof [288].

This variability amongst the majorgyre frame and subsumed gyrosystems yields an inordinate number of permutations to the individual units in the polymeric form, for example: [C^O^]_1_ models C^O^3e^^, which is CH_3_O, or methanol; [C][C^O^] models C^3e^C^O^3e^^, which is C_2_H_6_O, or ethanol; [C^O^]_3_ models C^O^3e^^C^O^2e^^C^O^3e^^, which is C_3_H_8_O_3_, or glycerol; and so on. Glycerol is a fundamental unit of any phospholipid and thus its origin is of great interest to biopoesists [289].

*Fatty acid*. The current chemical model for cellular fatty acid synthesis involves acetyl-coA and malonyl-coA precursors and proteins called fatty acid synthases [290,291]. The fundamental problem with this model from an evolutionary standpoint is that metabolic processes undergird the formation of RNA, which is required for creating any protein. An alternative pathway focused on chemical origins, where primitive fatty acid synthesis is dependent upon a glycoaldehyde substrate, has been proposed [292]. Given these differing views, I applied the 1°/3° alternacarbogyre to modeling simple and complex fatty acids. For example, the polymer C^2O^[C]_n_ is *any* fatty acid—where C^2O^ models a carboxyl “head” group (COOH; rather than carbon dioxide) and, for the [C]_n_ “tail,” for example, the [C]_1_ moiety is -CH_3_, acetic acid, an important molecule for the origin of life [293,294]; [C]_2_ is -CH_3_CH_2_, propionic acid, a nutritionally relevant fatty acid [295]; and [C]_13_ is -(CH_2_)_12_CH_3_ is myristic acid, a regulatory fatty acid [296,297]. Any other saturated or unsaturated fatty acid [298,299] in distinct microorganisms [300,301] can be similarly modeled. Given the emergence of glycerol and the nature of fractal Matrioshkagyre sets, any mono-, di-, and triglyceride [302] can likewise be positioned here.

*Asteroids and Comets*. The Solar System has an asteroid belt between Mars and Jupiter and another, the Kuiper belt, beyond Neptune’s orbit [303,304]. It has been argued that these and other asteroids are remnants of Solar System genesis—collisional break-up of large parental bodies [305]. One other class of prominent bodies is comets, whose origins are thought to be found in the Kuiper belt or in the Oort Cloud, a hypothetical cloud of icy bodies at the edge of the Solar System [306,307]. Because the electrogyre and oxygyre have cosmic applications—modeling the origin of planets (3.1) and moons (3.2), respectively—I thus applied the carbogyre to mysterious origins of asteroids and comets. Since asteroids and comets are carbonaceous and largely icy [308,309]—a comet is, by definition, 85% ice—this is consistent with the oxyon singularity at the core of the carbogyre. Moreover, the origin, evolution, and organization of the carbonates and carbonatites that comprise meteorites [310] and the Earth [311] are accounted for by attractorepulsion of the gyrapex of the carbogyre by the electrogyre. The gyrobases of the secondary and tertiary carbogyre models polyols as well, like those found in the Murchison meteorite [312]. Thus, the carbogyre is a broad-ranging model for understanding the physical properties and behavior of asteroids, comets, meteors, and other related celestial objects [313].

*From Organic Chemistry to Phosphochemistry.* How the living cell and planet Earth both are capable of producing and perennially reproducing their exquisite carbon chemistry and biochemistry in exactitude is modeled as ***carbognosis***—universal learning of organic compounds in response to adaptational cues derived from electron and oxyon flow—and ***carbomnemesis***—retention and retrieval of the ordering. Notably, these phenomena support the organic nature of the Earth found in the Gaia hypothesis [314,315,316].

The nesting of the photon within the electrogyre, the electron in the oxygyre, the electron in the carbogyre, and the oxyon in the carbogyre reveals how carbohydrates and hydrocarbons store energy and information that performs—and can be extracted to perform—specific physical, biophysical, and geophysical work. As found in almost all critical biometabolic pathways in the three kingdoms of life, the cycling of organic matter sustains and adapts the phosphochemical systems of life.

### 3.4. Phosphomembranes

All living cells have membranes composed of phospholipids that are necessary for compartmentalization of biometabolic processes [317,318,319]. All living cells use the high-energy phosphate bond for mobilizing energy from one molecule to another [320]. Phosphorus is also thought to be geochemically important to the evolution of early Earth [321]. How and why phosphorus has such a prominent role in both the structure and function of life is an unsolved matter.

In the search for the origin of life, paleobiologists have suggested that finding distinct membranous architectures in ancient rock layers is a key hint regarding the existence of life during geological epochs [322,323]. It is unclear whether the paleobiological findings reflect imprints of membranous compartments (coacervates [324], lipid vesicles [325], protocells [326]) filled with a metabolic soup, although certain additional biological signatures are also used to claim what is and is not a true fossilized cell. A scientific theory of life should inform whether membranous cavities emerged prior to dividing cells. In addition to addressing this matter, the ***phosphogyre*** models the origin and evolution of the high-energy phosphate bond that all living organisms on Earth use for storing and mobilizing chemical energy.

From the phosphogyre onward, the reader should appreciate that the gyromodel-defined quanta are polymers, systems, and molecular aggregates; this symbolic representation contrasts the quantized electrons, elements, and chemical molecules as outlined in the electrogyre, oxygyre, and carbogyre. For theoretical thrift and consistency, the phosphogyre does not symbolically disclose the oxyon, electron, and photon even though they are subsumed by it (G_VIII_).

*Phosphoenolpyruvate*. In reaction chemistry and biochemistry, the Gibbs free energy (∆G) is a value that represents the thermodynamic potential to do work [327,328]; the lower the value, the greater potential. The molecule with the highest chemical bond energy in life (∆G = −61.9 kJ/mol) is the versatile small molecule phosphoenolpyruvate (PEP) [329]. In the bacterial cell, PEP is the energy source for the phosphotransferase system [330]. So: How did PEP emerge in the evolution of life? Considering that the ***primary phosphogyre*** (Figure 2d (*i*)) is

P^3C^ ⇆ P^2C^ + Ⓒ

this fits the chemical reaction

C_3_H_5_O_6_P ⇆ C_2_H_3_O_5_P + CH_2_O

which shows interconversion between PEP (C_3_H_5_O_6_P) and acetyl phosphate (AcP; C_2_H_3_O_5_P) and formaldehyde (CH_2_O), the gyradaptive singularity—the carbyon that is rapidly polymerized ([C^O^]) in the gyrobase of the secondary carbogyre. Whereas AcP is a phosphate donor molecule with central roles in bacterial biosynthetic and nutrient sensing pathways [331,332], PEP is the penultimate component in glycolysis and the second component in gluconeogenesis [333]. Further evidence of the relationship of PEP and AcP to the oxygyre and electrogyre is found in the following oxidation-reduction reaction (a variant on the pyruvate oxidase reaction that oxidizes pyruvate (C_3_H_3_O_3_^−^) to acetate (C_2_H_3_O_2_^−^) and CO_2_ [334]):

pyruvate + P_i_ + O_2_ ⇆ AcP + CO_2_ + H_2_O_2_

Here, P_i_ is inorganic phosphate (HPO_4_^2−^).

PEP is also involved in another very important photosynthetic process in plants called C4 carbon fixation [335]. I can modify the longhand reaction

PEP + CO_2_ + H_2_O → oxaloacetic acid + P_i_

to

PEP + H_2_CO_3_ → OAA~P_i_

showing both the unstable carbonic acid and unstable transition state (~) phosphate molecule. OAA has the chemical notation of C_4_H_4_O_5_^2−^, the equation balances and can be written in the bidirectional, inverted gyrequation shorthand:

P^4C^ ⇆ P^3C^ + Ⓒ

This crucial step in C4 fixation is thus an alternagyre that oscillates between 4 carbyon (excited; gyrapex) and 3 carbyon (ground; gyrobase) states. I have depicted the carbonic acid-as-singularity in Figure 3d and have put the electrogyre, oxygyre, carbogyre, and this phosphogyre into a Matrioshkagyre (Figure 3e). Notably, OAA is a component in the citric acid cycle [336], and, being composed of C, H, and O, is effectively modeled in the secondary carbogyre. The carbyon-phosphogyre nesting is thus theoretically compatible with the empirical evidence.

Theory thus shows that, like the emergence of water (3.2) and carbon dioxide (3.3) before, phosphorous compounds emerge from the expansion of the universe from within the electrogyre, into and through the oxgyre and carbogyre, and forming a gyrosystem with relativistically lower exergy, opposing chirality, and evolutionary novelty (G_XIII_, G_VI_, G_IX_). Together, this expansive force, the inherent creatodestructive nature of the gyre, and the fact that ∆G is derived from the energy-rich photons housed in the electrons themselves (positioning PEP proximal to the gyradaptive singularity), cumulatively explain the origin of the high-energy phosphate bond.

*Phospholipids*. The biochemical and molecular structure, function, and regulation of cellular phospholipids is well known [337,338]. However, the field lacks a unifying framework. Here, the primary phosphogyre models the most basic of all phospholipids, where the gyrapical P^3C^ models 3-carbon (3C) glycerol “backbone” with a diglyceride “tail” and a 1-orthophosphate “head” group (P; additional carbyons, oxyons, and electrons are excluded for theoretical tidiness; note the triquantal organization). The cycling carbyon (Ⓒ; gyradaptor) models fatty acids (3.3) which, given G_I_, exerts the thermodynamic attractive force, or breakdown of the two-tail (P^3C^) phospholipid to a one-tail (P^2C^) state:

P^3C^ → P^2C^ + Ⓒ

and the countervailing repulsive force to construct the two-tail state:

P^2C^ + Ⓒ → P^3C^

Based upon this model, fatty acid uptake is biophysically “easy [339].” Lexically, one cycle of the carbyon particle through the phosphogyre is called a ***phosphocycle***.

Because the primary phosphogyre expands from the singularity omnidirectionally outwards, monolayer single-tailed phospholipids form micelles (see below for bilayered structures), spherical structures called here ***phosphospheres*** [340,341].

In the ***secondary phosphogyre*** (Figure 2d (*ii*)), two carbyons cycle through the singularity:

P^3C^ ⇆ [P^C^]+ 2Ⓒ

causing the gyrosystem dIEM to exist as either (G_V_) the high energy, learning state (the two-tailed phospholipid is a ***phosphognose***) or the low energy memory state (***phosphomneme***) with the potential to polymerize ([P^C^]; gyrobase). In the polymer, called a ***phosphonexus***, C is retained as the gyrolink (G_X_) that facilitates polymerization with P_i_ gyromodules:

[P^C^] = P^C^, P^C^P^C^, P^C^P^C^P^C^, P^C^P^C^P^C^P^C^P^C^….

I want to call attention to what theory predicts: the phospholipid head group separates from its two-tail group, leaving an “untailed” head group consisting of the C1 of glycerol and the orthophosphate and a “headless” C2 and C3 of glycerol with the tail group of acyl chains. In a phosphonexus, one carbyon gyrolinks two flanking ***phosphons*** (quantized particle (Figure 3d (*iv*)) known as two monoester bonds (oxygyre). Membrane fluidity—which has been understood largely from the fluid mosaic model [342,343]—can now be clarified as phosphonexus structure and metabolism, or oscillation between the anabolic state generated by the attractive force of the dicarbyon,

P^3C^ → [P^C^]+ 2Ⓒ

and the catabolic state, repulsion by the dicarbyon that regenerates the phospholipid,

[P^C^] + 2Ⓒ → P^3C^

The innate adaptability of the phosphogyre explains homeoviscous adaptation [344]. Fluidity is also better understood as the spacetime path of the carbyon in its orbit around the phosphon in the phosphonexus. The unitary P^C^ accommodates at least two different chemistries: (*i*) methyl phosphate (MeP), a phosphoryl transfer molecule with a very fast rate of hydrolysis (oxygyre attraction (3.2); [345,346]) and (*ii*) carboxyl phosphate, which is a short-lived biochemical intermediate [347]. Oxygyre repulsion would model condensation and hence polymerization ([P^C^]). Longer phosphonexuses are predicted to be in a levoral organization (Table 2) called ***phosphohelices*** that toggle between the two chiralities (G_XII–3_) as they structurally complexify*.* These phosphonexuses and phosphohelices verifiably assemble into ***phosphomatrices***, a layered architecture that, in effect, “floats” on top of the headless dicarbyon.

With this model, I can now explain several enigmatic features of phosphomembranes. First, the floating phenomenon models what has been described in the literature as lipid rafts [348], as surface areas move as an ensemble. Second, given the repulsive nature of the oxyon on the tertiary carbogyre (3.3), the acyl chains align and appose, modeled as the antiparallel flow of one phosphogyre along another—one phosphogyre exerts a countervailing force against the other, providing instrinsic and extrinsic balancing, *i.e.* “cross talk” between the two layers [349]. This antiparallel structure represents the phospholipid bilayer that is found in all cell membranes and in organelles (e.g., Golgi apparatus, endoplasmic reticulum, peroxisome, vacuole [350,351,352,353,354,355,356]). Third, because an individual acyl chain can be metabolized by the singularity in either one of the two antiparallel carbogyres, this explains lipid diffusion and spontaneous fatty acid flip-flop [357,358]. Fourth, the inherent adaption of the phosphogyre explains the balance of phosphate chemistry within and without of a membrane compartment, called phosphate homeostasis [359,360]. Fifth, phase shifting to and from the membrane solid state [361] is explained by phosphogyre arrest and release, respectively—changing photon (energy) flow through the electrogyre, oxygyre, and carbogyre controls phosphon cycling (***phosphocycling***) between gyrostates.

If P^3C^ is modeled to be the two-tailed phospholipid and [P^C^] is phosphonexus, then what is the gyradaptive 2Ⓒ? As fit to the gyromodel, 2Ⓒ corresponds to cholesterol [362], members of a family of sterol molecules that are involved in numerous signaling cascades [363], vitamins [364], coenzymes [365,366], flavonoids [367], and tocopherols [368]. These compounds emerge in the carbogyre and exert strong thermodynamic forces as the gyradaptive singularity of the phosphogyre.

*Polyphosphate*. All kingdoms of life have inorganic polyphosphates that can range in length from a few orthophosphates to several hundred long. These polyphosphates have been implicated in a variety of essential biological phenomena including, but not limited to energy storage, biofilms formation, stress-induced gene regulation, cell motility, virulence, cellular proliferation, differentiation, and development [369,370]. For these and other reasons, Kornberg asserted that polyphosphate is of genuine theoretical interest as a prebiotic precursor to RNA, protein, and DNA [371].

I have applied the empirical evidence about polyphosphates to one of the majorgyres of the gyromodel, the ***tertiary phosphogyre*** (Figure 2d (*iii*)). This gyrosystem shows the extreme cycling of the tricarbyon (3Ⓒ; gyradaptor) which models a monoglyceride [372,373], diacylglycerol—an established second messenger signaling lipid [374]—or any of a number of trigylcerides [375,376]. The attractive force of the tricarbyon on the phosphogyre elicits the formation of a phosphonexus:

P^3C^ → [P]+ 3Ⓒ

Given G_XI_, the gyrobasal [P] has a oxyon gyrolink and models: orthophosphoric acid ([P]_1_ = P^O^; H_3_PO_4_), the hallmark of all protein signal transduction cascades [377]; pyrophosphoric acid ([P]_2_ = P^O^P^O^; H_4_P_2_O_7_), found in all nucleic acid polymerization reactions (3.5 and 3.7) and numerous intracellular and extracellular processes [378,379]; triphosphoric acid ([P]_3_ = P^O^P^O^P^O^; H_5_P_3_O_10_), one of three major parts of the nucleotide triphosphates (3.5); and polyphosphoric acid ([P]_n_ = P^O^P^O^P^O^P^O^P^O^…). Notably, polyphosphates assemble into helices (Figure 4d; [215,380]). The catabolism of these polymers is modeled vectorially as:

[P] + 3Ⓒ → P^3C^

where tricarbyon generates the PEP or phosopholipid molecule. In this regard, another notable reaction can be neatly fit onto the tertiary phosphogyre:

PEP ⇆ P_i_ + pyruvate

The relationship between the three majorgyres can be equated through the shared gyrapex,

P^2C^ + Ⓒ ⇆ [P^C^] + 2Ⓒ ⇆ [P]+ 3Ⓒ

balancing, I have:

P^2C^ ⇆ [P^C^] + Ⓒ ⇆ [P]+ 2Ⓒ

This gyrequation provides a parsed relationship between phosphochemical energy storage and lipid signaling pathways in all cells, which, written longhand, can be:

one-tailed phospholipids ⇆ phosphonexuses + fatty acids ⇆ polyphosphates + sterols.

Other phosphorous compounds that are important to the evolution of the early Earth are modeled here [381,382,383,384].

*Biogeochemistry*. The phosphorus cycle is one of the major biogeochemical cycles that occurs on Earth and is essential for life [385,386,387]. Together, the thermodynamic relationships from the electrogyre (3.1) to the oxygyre (3.2), carbogyre (3.3), and phosphogyre capture elemental, inorganic, aqueous, and organic states of the phosphorus cycle. Like all other biogeochemical cycles that are viewed in four dimensions, the phosphorus cycle is a ***macrophosphogyre***.

*From Phosphochemistry to Genetic Information.* I have shown the phosphogyre to have broad explanatory power. Given that there is no general theory of phosphate biochemistry, the phosphogyre affords unparalleled insight into fundamental characteristics of all life on Earth. Given that all cellular phosphate molecules are modeled to undergo phosphognosis and phosphomnemesis in response to cues from sub- and supervenient gyrosystems, this supports the concept of membrane heredity [388]. Moreover, the phosphogyre helps clarify the omnidirectional phospholipid façade of the cell, membrane-bound organelle inheritance [354], functional symmetry of endomembranes [389], and the establishment and maintenance of its phosphate-dependent signal transduction cascades. Regarding the evolution of life, the nested gyrosystem architecture demonstrates that phospholipid structures formed prior to nucleic acids, proteins, and dividing cells, consistent with hypotheses related to minimum protocell evolution [390] and the lipid world model [391].

At this point, I have a framework that seamlessly integrates phosphate (P) and carbon (C, carbogyre) chemistry, oxygen (O, oxygyre), and hydrogen (H, primary electrogyre), or CHOP. However, because life is ~98% CHNOPS, I require models that fit the scientific data regarding biomolecules composed of nitrogen (N) and sulfur (S). In knowing that orthophosphate is a core component of all nucleic acids and being practiced in theoretical RNA biology [392], I modeled the phosphon as the thermodynamic driving force for the emergence of nucleotides and genetic information.

### 3.5. RNA

The RNA molecule that transmits genetic information is an essential feature of all life. Current notions for how cells are genetically regulated are derived from Crick’s central dogma [393]. This dogmatic model specifies the following: (*i*) DNA is the long-term, stable genetic storehouse; (*ii*) DNA is a template for messenger RNA (mRNA), a short-lived molecular go-between; (*iii*) mRNA, along with ribosomal RNA (rRNA) and transfer RNA (tRNA), assemble amino acids into polypeptides (proteins). This parsed linear flow of genetic information is

DNA → RNA → protein

where the arrow between DNA and RNA is the process called transcription and that between RNA and protein is called translation.

Several pieces of evidence and investigators have called this model into question [394,395,396] and have implied a more primal role for RNA than originally thought. Gilbert was the first to formally promulgate the RNA world hypothesis, in which RNA emerged evolutionarily prior to DNA and protein [397]. Although his radical proposal is compatible with the available data [7,8,9], there is no genetic theory that includes and validates the hypothesis. On this note, the ***ribogyre*** is a theoretical framework for understanding the emergence, adaptation, and metabolism of genetic information.

*Nucleotide triphosphate*. How the nucleotide originated is one of the more challenging biosynthetic enigmas [398,399,400,401]. Here, I fit the well-known biochemical reaction:

NTP ⇆ NDP + P_i_

where NTP and NDP are the nucleotide tri- and diphosphates, respectively, and P_i_ is the gyromodule of the tertiary phosphogyre (3.4), onto the ***primary ribogyre*** (Figure 2e (*i*)). That reaction, as a gyrequation, is:

R^3P^ ⇆ R^2P^ + Ⓟ.

R corresponds to the grouped nucleotide sugar and nitrogenous base and gyradaptive P/Ⓟ is the particle/quantum orthophosphoric acid, the phosphon—the mIEM and singularity of the ribogyre. Given the protean quality of gyromodel symbolism, the gyrapical R^3P^ and gyrobasal R^2P^ can represent any one or all NTPs (adenosine triphosphate (ATP), guanosine triphosphate (GTP), cytidine triphosphate (CTP), and uridine triphosphate (UTP)) and NDPs, respectively.

In the ribogyre, NTP generation is modeled as the repulsive force of the phosphon on an NDP molecule itself:

R^2P^ + Ⓟ → R^3P^

Conversely, NTP catabolism is modeled as the attractive force of the phosphon:

R^3P^ → R^2P^ + Ⓟ

The primary ribogyre thus accounts for all NTP/NDP cycles required to establish energy and matter gradients in cell regulation and signal transduction pathways [402,403],

ATP ⇆ ADP + Ⓟ

vesicle [404] and cargo [405] transport,

GTP ⇆ GDP + Ⓟ

sugar [406,407] synthesis;

UTP ⇆ UDP + Ⓟ

and lipid [408] synthesis,

CTP ⇆ CDP + Ⓟ

Please note the triquantal feature of the NTP molecule: nitrogenous base (tertiary ribogyre, see below), ribose (secondary carbogyre), and triphosphate (tertiary phosphogyre). Additional carbyon-ribogyre relationships facilitate modeling molecules called nucleotide sugars [409].

I call attention to four gyraxioms as they relate to this gyrosystem. First, the generation of the ATP in the ribogyre is consistent with the dependence of subsumed gyrosystems (G_VIII_) and with the chemiosmotic hypothesis [410]—it is thermodynamically dependent upon electron mobilization (electrogyre), in redox reactions (oxygyre), through organic matter (carbogyre), in a phospholipid membrane (phosphogyre), onto water (oxygyre), with deposition of accumulated potential energy into orthophosphate (phosphogyre), on a nucleotide (ribogyre). Second, a nucleotide can exist in either NTP *or* NDP form but cannot exist in both states at the same time (G_V_). Third, it is also known that nucleotides are exclusively dextral in life—L-nucleotides are unnatural and cause significant structural alterations to nucleic acid structures [411]—but it is not clear why. The chirality of the nucleotide is dextral because the phosphogyre is dextral (G_XII_). Fourth, the ribogyre, in emerging from the phosphogyre, dictates that ATP is relativistically less exergic but more stable (G_XIII_) than pyrophosphate and also more evolved [412,413].

*Transcription and turnover*. The idea that RNA is “transcribed” from—that is, copied or templated from—DNA is deeply engrained in the scientific literature [414,415,416]. However, the origin of novel small RNA species [417,418], changes to or rearrangements in RNA sequence [419,420], intronic origins [421], and different RNA turnover rates [422] have not been adequately explained by the DNA-centric RNA biogenesis idea. I thus determined how the majorgyre frame could resolve these discrepancies.

The *secondary ribogyre* (Figure 2e (*ii*)) models the metabolism of RNA:

n × NTP ⇆ RNA + n × P~P

where P~P is pyrophosphate and n = any positive integer. This precisely fits the gyrequation:

R^3P^ ⇆ [R^P^] + 2Ⓟ

where, again, R^3P^ is NTP, the shared gyrapex of the majorgyres, 2Ⓟ is pyrophosphate or two orthophosphates, and [R^P^] is a nucleotide monophosphate (NMP) with the potential to polymerize into RNA, referred to here as a ***ribonexus***. For example,

R^P^, R^P^R^P^, R^P^R^P^R^P^, R^P^R^P^R^P^R^P^R^P^R^P^…

is the same as

mononucleotide, dinucleotide, trinucleotide, and hexanucleotide.

Note that, in the ribonexus, the phosphon (P) is the gyrolink and the mononucleotides (R) are the gyromodules (G_X_).

Now, with this new understanding, transcription (RNA “expression”) is modeled vectorially as diphosphon attraction,

R^3P^ → [R^P^] + 2Ⓟ

and RNA turnover is modeled by the repulsive force of the diphosphon,

[R^P^] + 2Ⓟ → R^3P^

with ribonexus disassembly into component nucleotides that are restored to the gyrapical state.

The closer the ribonexus is to the gyradaptive diphosphon, the faster it cycles between the two gyrostates; the further from the singularity, the slower it cycles. The secondary ribogyre thus affords a new perspective on the varying RNA half-lives and cellular transcription cycle (***ribocycle***; [423,424])—where viewing in four dimensions reveals the gyre. I present the diphosphon-as-singularity concept in Figure 3f. Combining, compressing, and reducing the primary and secondary ribogyres yields:

R^2P^ ⇆ [R^P^] + Ⓟ

which validates the interconversion of NDPs and NMPs and both confirms and predicts a basic biometabolic relationship between NDPs and RNAs [425,426]. The cycling of the full complement of RNAs (transcriptome) in a cell, organism, or species in an ecosystem or planet occurs within a ***macroribogyre***.

*RNA Structure and Function.* RNAs continually adapt and evolve through a process known as **ribognosis**, whereby gyrapical NTPs import and integrate information related to the phosphochemical energy state of membrane compartment (phosphon). The gyrobase of the ribogyre, being a **ribomneme**, stores information about nucleotide content for *all* RNA classes, especially the three main classes: mRNA [427,428], tRNA [429,430], and rRNA [431,432]; I return to these in 3.6. Similar to other gyronexuses, ribonexuses form higher order structures theoretically defined as **ribohelices** (e.g., stem-loops [433], hairpins [434]) and **ribomatrices** (Figure 4e; splicing RNAs [435], rRNA complexed with mRNA and tRNA [436]) that toggle between chiralities as they complexify (G_XII–3_).

*Secondary messengers and cofactors*. Given its symbolic depth and intrinsic gyrosystems, R^P^ models cyclic AMP (cAMP; [437,438]) and cGMP [439]; [R^P^]_2_ models dinucleotide molecules (e.g., cyclic di-GMP [440,441], nicotinamide adenine dinucleotide (NAD; [442])). Other nitrogenous cofactors are positioned here [443,444].

*Genetic code*. The origin of the genetic code is one of the leading problems in evolutionary biology [445] and thus pinning down this problem requires a ground head chancery. Since majorgyres dictate that energy and matter assemble into triquantal (most exergic, least stable), diquantal (intermediate energy and stability), and uniquantal (least exergic, most stable) states (2.4.5), I applied this concept to understand the organization of genetic information. As modeled *sans* proteins, within the ribonexus, [R^P^]_3_, the trinucleotide—rather than the mononucleotide as used by polymerases [446,447]—is the high-energy triquantal unit that polymerizes, where

3R^3P^ ⇆ [R^P^]_3_ + 6Ⓟ, and

[R^P^]_3_ = R_ξ_^P^R_ψ_^P^R_ζ_^P^

and R_ξ_^P^ is first nucleotide of the codon, R_ψ_^P^ is second nucleotide, R_ζ_^P^ is third nucleotide. The dynamics of this gyrosystem can also be visualized via two gyrequations:

R_ξ_^P^R_ψ_^P^R_ζ_^P^ ⇆ R_ξ_^P^R_ψ_^P^ + R_ζ_^P^

R_ξ_^P^R_ψ_^P^R_ζ_^P^ ⇆ R_ξ_^P^ + R_ψ_^P^R_ζ_^P^

Although the triribonexus (R_ξ_^P^R_ψ_^P^R_ζ_^P^; triquantum) can be modeled in the secondary ribogyre, here, the R_ζ_^P^ or R_ψ_^P^R_ζ_^P^ are the gyradaptive force of an auto-assembling, auto-adaptive, auto-metabolic alternagyrosystem. The trinucleotide is the most unstable and susceptible to change, the dinucleotide (R_ξ_^P^R_ψ_^P^; diquantum) is relativistically more stable, and the mononucleotide (R_ξ_^P^; uniquantum) is the most stable. Since the 3^rd^ nucleotide has the broadest genetic information flexibility and the 1^st^ is the most constrained vis-à-vis its encoded amino acid [448], this triquantal organization concomitantly evinces an organizational basis for the triplet codon and intimates its degeneracy and the basis of the wobble [449]. I expand on the specificity of the code in 3.6.

*Ribovirogenesis*. Whether or not viruses are alive is a matter of much debate and speculation [450]. Furthermore, while there are many ideas related to how viruses evolutionarily originated [451,452,453], there is no consensus model. Given the emergence of genetic information (ribogyre) within a phospholipid bilayer (phosphogyre) in the absence of cell division (3.8), the ribogyre parsimoniously models modern ribovirogenesis—including retroviruses like human immunodeficiency virus [454]—or the origin and evolution of primitive RNA viruses. Notably, many viral RNA assemble as a spherical structure [455], known here as a ***ribosphere***. Finally, the RNA virus life cycle, when viewed in four dimensions, is revealed to be a ***ribovirogyre***.

*Nucleotides and Nitrogenous Compounds*. The ***tertiary ribogyre*** (Figure 2e (*iii*)) represents the cycling of a gyradaptive triphosphon (3Ⓟ), modeling the origin, evolution, and existence of a pool of, sets of, or individual nucleosides, nitrogenous compounds, or nitrogenous bases (R) [456,457]. The predicted polymeric signature with the sub_2_gyre gyrolink (as per G_XI_) is identifiable by linearizing the ring structure of adenine:

[R] = N^C^N^C^N^C^N^C^N^C^

where N is the nitrogen gyromodule and C is the carbyon gyrolink. The tertiary ribogyre is written in gyrequation form as

R^3P^ ⇆ [R] + 3Ⓟ

which, written elementally (excluding trace but physiologically important elements), models

CHNOP ⇆ CHON + P

Molecules that are positioned in the gyrobase of the tertiary ribogyre retain the nitrogen but lack the compositional and thermodynamic signature of the phosphon. These CHON biomolecules are antioxidants like melatonin [458,459], catecholamines [460], nitrogen heterocycles [461], coenzymes [462], tetrapyrroles [463], xanthines [464], folic acid [465], urate [466], serotonin [467], sphingosine and ceramide [468], and, importantly, amino acids. To these I turn.

*Origin and Homochirality of Amino acids.* There is no general theory to explain the origin and evolution of amino acids, although the Miller-Urey experiment [469] is frequently cited as a means for their generation. As just alluded, eighteen of the twenty common amino acids have CHON composition, and are thus are modeled as undergoing metabolism in the tertiary ribogyre: electron- and oxyon-mediated catabolism of the carbyon (ribose sugar) and ***ribon*** (nitrogenous base; the identifier of the ribogyre as a quantum or particle, Figure 2e (*iv*)) into linearized, branched molecules. Consistent with their positioning in the tertiary ribogyre, glycine, glutamine, glutamate and aspartate are implicated in the biosynthetic origin of purine and pyrimidine rings [470,471,472]. Gyrosystem breakdown of guanine at the carbonyl gives

N^C^(N)N^C^N^C^**N^CC^O^^**

where (N) is a branched nitrogen bond, and the last four atoms (in bold) are identical to the amino acid backbone with the amino head group, internal carbon, and carboxy terminus. Electron- and oxyon-mediated remodeling of uracil and cytosine has potential to generate certain amino acid side groups [473]. The amino acid histidine [474] bears the signature of the nitrogenous base.

The homochirality of amino acids is dispatched by one gyraxiom: IEM that emerges in or is modeled by a tertiary majorgyre spins in the direction of the sub_2_gyre (G_XII–2_). In other words, because the carbogyre is a levoragyre, and amino acids are positioned in the tertiary ribogyre, amino acids exist almost exclusively in the L-form.

*Biogeochemical Nitrogen Cycle*. Earth’s atmosphere is ~80% N_2_. How this came to be is not necessarily clear, although the biogeochemical cycle of nitrogen is indubitably an important aspect of the Earth system [475] and required for the existence of life. Given long-range thermodynamic interaction through the tertiary majorgyre gyrobase (G_XI–1_), the tertiary ribogyre is the entry point for the biogeochemical nitrogen cycle, with atmospheric nitrogen, N_2_, and its fixation to NH_4_^+^ modeled by autocatalysis of the electrogyre, conversion to nitrites and nitrates [476] modeled by the thermodynamic repulsive force of the oxygyre, and assimilation modeled by thermodynamic repulsion by the carbogyre on the nitrogen into amino acids (e.g., aspartic acid, glutamic acid, glycine, alanine, and arginine [477]). Stepping back to the electrogyre and oxygyre, nitrogen cycling can now be modeled as its own set of majorgyres, called *nitrogyres*:

Primary nitrogyre: NO_3_^−^ ⇆ NO_2_^−^ + O (N^3O^ ⇆ N^2O^ + Ⓞ)

Secondary nitrogyre: NO_3_^−^ ⇆ NO + O_2_ (N^3O^ ⇆ [N^O^] + 2Ⓞ)

Tertiary nitrogyre: NO_3_^−^ ⇆ N + O_3_ (N^3O^ ⇆ [N]_1−n_ + 3Ⓞ)

Nitrate (NO_3_^−^), nitrite (NO_2_^−^), and nitric acid (NO) are reactive and important inorganic biochemicals [478,479]. NO is a biochemical component of L-arginine metabolism [480], additional confirmation that the tertiary ribogyre fits amino acids. In addition to these reactive chemicals, the tertiary nitrogyre gyrobase has three major forms (other allotropes exist as well, all of which are unstable): N_3_, represents azide, an amine precursor [481] that is highly reactive and unstable (an explosophore); N_1_ corresponds to elemental nitrogen (modeled by the electrogyre), which by virtue of its trivalence (another triquantal form) rapidly forms N_2_. The nitrogyre thus represents, models, and explains the N_2_ and other fundamental nitrogenous compounds that accumulate(s/d) on Earth [482,483].

*From RNA to Polypeptides.* The ribogyre shows how life uses nucleotides for both information transmission and energy storage. Furthermore, the ribogyre validates the existence of an RNA world prior to the emergence of protein, DNA, and the living cell. Although ribozymes (catalytic RNAs molecules [484]) have been proposed to play an important role in the RNA world [485], this theory shows an alternate view for ribogenesis. One important ribozyme, rRNA [486], catalyzes amide bond formation in protein synthesis and, along with mRNA and tRNA, represents the thermodynamic driving force for the emergence of the next gyrosystem in the evolution of life.

### 3.6. Protein

Despite a great deal of hypothesizing about the origin of the translation complex [487,488], there has not been one idea or model to gain wide scientific approval. What is agreed upon is that polypeptides emerge from *within* a macromolecular complex of RNAs called the translation apparatus. This nested organization is noteworthy, as it permits the consistent modeling of the ***aminogyre*** as emerging from within the ribon.

Before I continue, two points. First, the symbol R refers to, for example, a unique RNA molecule, a pool of the same class of RNAs, an RNA complex, nucleotide, nucleoside, nitrogenous base, and/or the amine group that defines each of these molecules. In other words, the ribon captures a wide range of macromolecules and chemistries, whose identities may be lost to the unified symbolism of majorgyres. Second, the aminogyre is a gyromodel that simultaneously fits data related to a polypeptide’s evolution on Earth and its present-day functions in the cell.

*Specificity of the Genetic Code.* Understanding the specificity of the genetic code (introduced in 3.5) requires a deconstruction of the ***primary aminogyre*** (Figure 2f (*i*)), where

A^3R^ ⇆ A^2R^ + Ⓡ

is rewritten as

A^R´R´´R´´´^ ⇆ A^R´R´´^ + R´´´, and

R´ = mRNA(s)

R´´ = tRNA(s)

R´´´ = rRNA(s), and

A = amino acid(s) (aa; or amino acid polymer)

Substituting into the gyrequation, I arrive at the following schema:

aa-tRNA/mRNA/rRNA ⇆ aa-tRNA/mRNA + rRNA

where aa-tRNA represents charged aminoacyl-tRNAs ([489]; see below). As with other gyrapices, A^R´R´´R´´´^ is unstable. By comparison, the gyrobasal A^R´R´´^ is relativistically more stable, modeling the pool of stably aminoacyl-charged tRNA and mRNA in a ternary complex. (Note that A^2R^ has potential to model A^R´R´´´^, A^R´´R´´´^, or any other two RNA species.) The gyradaptive ribon (Ⓡ) depicts rRNA (or any RNA species that impacts the structure of the gyrapical complex). Written another way, ribon repulsion elicits quarternary complex formation,

A^R´R´´^ + Ⓡ → A^R´R´´R´´´^

and ribon attraction evicts an adapted ternary complex,

A^R´R´´R´´´^ → A^R´R´´^ + Ⓡ

The primary aminogyre thus demonstrates that the aminoacyl-tRNA and mRNA physically co-adapt. The co-gnostic and -mnenomic shaping of these ribonucleotide classes is detectable in codon and anti-codon identity and amino acid specificity (***aminognosis*** and ***aminomnemesis***). Further, the primary aminogyre predicts that this co-adaptational process proceeds vectorially through the rRNA particle—but without amide bond formation. This model is compatible with the co-evolution theory of the genetic code [490,491]. Finally, as there are three defined tRNA occupancy sites (A, P, and E [492,493]) in the rRNA, this reveals how amino acids, like the code and the NTP itself, are organized triquantally.

Since gyrequations permit themselves to representing the Matrioshkagyre organization (2.4.1), I can replace the ribons with their specific ribogyre and phosphogyre equations:

mRNA, tRNA, and rRNA = R^3P^ ⇆ [R^P^] + 2Ⓟ

18/20 amino acids (A; see below) = R^3P^ ⇆ [R] + 3Ⓟ

Substituting appropriately, the primary aminogyre could be thought of as:

(R^3P^ ⇆ [R] + 3Ⓟ)^3(R^3P^ ⇆ [R^P^] + 2Ⓟ)^ ⇆ (R^3P^ ⇆ [R] + 3Ⓟ)^2(R^3P^ ⇆ [R^P^] + 2Ⓟ)^ + (R^3P^ ⇆ [R^P^] + Ⓟ)

but even this representation excludes information. Nevertheless, with this provisional picture, I show that changes in orthophosphate levels impart changes in nucleotide salvage pathways [494] which, in turn, impart changes on RNA levels and composition and the metabolism of amino acids, which ultimately translates to the specificity of the genetic code.

*Sulfated Amino Acids and Biogeochemical Sulfur Cycle*. The vast majority of the sulfur in the living cell is found within polypeptides as cysteine and methionine [495]. Rounding out the positioning of biogeochemical cycles [496], I model the aminogyre as the input point for the sulfur cycle [497,498]: mineralization of organosulfur compounds and metabolism of elemental sulfur to H_2_S, hydrogen sulfide [499], is modeled as a consequence of the repulsive electrogyre; oxidation to HSO_4_, sulfate [500], is modeled by the repulsive oxygyre; sulfur assimilation to organic and nitrogenous sulfhydryl [501]—with sulfur being metabolized into the amino acids cysteine (Cys), homocysteine, methionine (Met), and taurine [502,503]—is modeled by the creatodestructive, expansocontractive, and attractorepulsive forces of the carbogyre through the tertiary ribogyre and into the aminogyre. The cycling of the full complement of amino acids, peptides, and proteins (proteome) within, between, and among cells in the biosphere is called the ***macroaminogyre***.

Positioning Met and Cys in the aminogyre abides by G_IX_—a novel IEM must emerge in a focagyre—since macromolecules containing the element sulfur emerge in the evolution of life. Notably, Met in particular initiates the polypeptide [504], indicating the evolutionary necessity for novel IEM. Finally, given that the ribogyre is a levoragyre, it exerts a left-handed chemosynthetic force (G_XII_) on Met and Cys; thus these amino acids are exclusively L-form in living systems. This theory eliminates the problem of homochirality of all amino acids, given aminogyre emergence from the phosphogyre and ribogyre; moreover, it is compatible with ideas of phosphate- and RNA-dependent mechanisms for generating amino acid chirality [505,506].

*Protein synthesis and degradation*. An accurate theory of life must have an explanation of how polypeptides are created and how they are destroyed. The current models to explain these phenomena are largely unrelated: at its core, protein synthesis involves a quarternary complex of mRNA, aa-tRNA, and rRNA [507] and protein turnover involves either the autophagic-lysosomal pathway [508] or targeting by specific protein enzymes or multi-protein complexes called proteases (e.g., the proteasome [509]). I thus applied the aminogyre framework to unify these processes. The ***secondary aminogyre*** (Figure 2f (*ii*)) is written as

A^3R^ ⇆ [A^R^] + 2Ⓡ

which models:

aa-tRNA/mRNA/rRNA ⇆ aa-tRNA + mRNA/rRNA

As shown above, the simplest unit is aminoacyl-tRNA. However, as G_X_ dictates—*i.e*., in a gyronexus, the gyrolink R is the dIEM of the subgyre—R corresponds to the amide bond (N) that links amino acids:

[A^R^] = A^R^A^R^, A^R^A^R^A^R^, A^R^A^R^A^R^A^R^A^R^A^R^…

which is

[aa^N^] = aa^N^aa^N^, aa^N^aa^N^aa^N^, aa^N^aa^N^aa^N^aa^N^aa^N^aa^N^….

Based upon this model, the gyrolink imports the information and energy from the tRNA (see 3.5); alternatively, nitrogenous bases impute genetic information into the amide bond. Making the gyrequation unidirectional, protein synthesis is modeled as diribon attraction,

A^3R^ → [A^R^] + 2Ⓡ

wholly consistent with release of the nascent polypeptide—called here an ***aminonexus—***from the rRNA and mRNA, with tRNAs displaced upon amide bond formation. Protein turnover, in contrast, is modeled as gyradaptive repulsion by the diribon,

[A^R^] + 2Ⓡ→ A^3R^

In other words, this theory of life predicts that RNA, nucleotides, organic bases have prominent and direct roles in protein metabolism. This protease-independent model of protein structure remodeling is consistent with data suggesting that changing levels of water (oxygyre), hormones and fatty acids (carbogyre), phosphorylation (phosphogyre), and amino acids (ribogyre) directly impact protein stability [509,510,511,512,513]. This model also provides a unique perspective on polypeptide evolution: ribon (nucleotides, RNA, amine)-based cycling through and into the aminonexus (***aminocycle***) allows the generation and feedback of domains and enzymatic activities into subgyres. In other words, theory explains the origin, evolution, and structure of novel protein motifs, domains, and folds [514,515]. Moreover, the proximity of the aminonexus to the diribon singularity determines its rates of turnover and evolutionary change [516]. Combining and compressing the primary and secondary aminogyres shows that

A^2R^ ⇆ [A^R^] + Ⓡ

an alternagyre that permits modeling of aminognosis of any two ribons (2R)—mRNA and tRNA, mRNA and rRNA, tRNA and rRNA, or any other RNA classes, species, or elements—with a polypeptide or amino acid (A). This 1°/2° alternagyre, along with the primary and secondary aminogyre (and subsumed gyres), provides a framework for understanding the origin of the translation apparatus in the evolution of life and additional modes of specificity of the genetic code.

*Aminoacyl-tRNA Metabolism*. To this point, I have not explained the origin and emergence of aminoacyl-tRNAs. Most current ideas related to their existence invoke aminoacyl-tRNA synthetases, proteins that attach an amino acid to a cognate tRNA [517,518]. Since this class of enzymes cannot emerge without translation, there is a chicken-and-egg enigma. Addressing this puzzle, by equating the secondary and ***tertiary aminogyre***s (Figure 2f (*iii*)), I have,

[A^R^] + 2Ⓡ ⇆ A^3R^ ⇆ [A] + 3Ⓡ

which, following compressing and balancing, is the 2°/3° alternagyre,

[A^R^] ⇆ [A] + Ⓡ

Based upon sub_2_gyre tertiary majorgyre gyrolink (G_XI_), the gyrobasal [A]_1_ is, for example, one or a pool of individual aa with a phosphon (aa~P), which represents

aa-tRNA ⇆ aa~P + tRNA

Thus, theory shows that in the evolutionary absence of the aminoacyl-tRNA synthetases, the ribon is the attractorepulsive force responsible for both creation and destruction of aminoacyl-tRNAs.

*Higher-order Protein Structure*. Similar to other gyronexuses, polypeptides assemble into α-helices ([519]; Figure 4f), 3_10_-helices [520], ∏-helices [521], β-sheet helices [522]—largely D-form to homeostatically balance the L-amino acids (G_XII–3_; Figure 4f). These structures are theoretically unified in the term ***aminohelices***. Aminohelices assemble into coils, fibers, and aggregates [523] that are termed ***aminomatrices***; these exist both intracellularly (e.g., higher-order micofilaments [524], microtubules [525], intermediate filaments [526]) and extracellularly (e.g., collagen [527], fibronectin [528,529], and laminin [530]). Finally, polypeptides assemble into aminomatrices that are architecturally spherical or ovoid such as a viral capsid [531] and clathrin cage [532]; these structures are ***aminospheres***.

*Protein Folding*. Anfinsen’s classic experiment—in which a denatured ribonuclease refolded properly, restoring catalytic activity [533]—led to many questions about how an unfolded polypeptide “remembers” its structure. Anfinsen himself suggested that the primary amino acid sequence determines native structure [534], but this idea doesn’t explain how a primary sequence *initially* acquires its folded state. This so-called protein folding problem [535] is resolved by this theory. Given the gnostic and mnemonic properties of the gyromodel (2.4.4), each of these gyrosystems [536] learns a particular spatiotemporal orientation, contextualization, and function, and, once stored and templated, remembers and restores it under appropriate thermodynamic conditions.

*Nucleoproteins and Post-translational Modifications.* The secondary aminogyre accounts for the origin and emergence of three classes of aminonexuses vital to nucleotide biochemistry: (i) nucleotide sensor enzymes (e.g., ribonucleotide reductases [537]); (ii) nucleotide-modifying enzymes (e.g., protein kinases and phosphatases [538,539,540], and DNA and RNA polymerases [541,542,543,544], nucleases [545,546,547], helicases [548]); and (iii) nucleotide-binding proteins (sequence-specific (e.g., transcription factors [549]) and sequence–nonspecific (e.g., histones [550]). I return to these three classes in 3.7. Polypeptides undergo different types of modifications [551,552,553,554,555,556]; the theoretical framework fits these as well [557].

*Phosphoproteins, Ribonucleoproteins, and Membrane Proteins*. Recall that a particle has quantum potential (G_III_), meaning that R has the potential to represent many distinct molecules, as does A. Because the phosphon is the gyrolink of the tertiary aminogyre (G_XI_), the 2°/3° alternagyre accounts for three distinct properties and characteristics of polypeptides. First, in the gyrapex, because phosphons in nucleotides (NTP, NDP, NMP) interact with ***aminon***s ((Figure 2f (*iv*); quantized particle that is the aminogyre (G_I_)), this models the establishment, maintenance, and chemosensory qualities of a nucleotide binding motif [558]. Second, aminonexus binding to ribonexuses is modeled here as well: [A^R^]_n_ represents these RNA-protein complexes called ribonucleoproteins, where [A] is one or more polypeptides that a gyrolinked by phosphates (e.g., post-translational modifications, sugar~P, or polyP) gyrobasally:

ribonucleoprotein ⇆ phosphoprotein + RNA

This schema fits numerous ribonucleoprotein complexes [559,560,561,562,563,564,565,566,567]. Phosphoproteins can also be viewed from the standpoint of the triribon singularity (Figure 3g). Third, the tertiary majorgyre facilitates a new understanding of polypeptide-phosphomembrane organization. Given that

[A] = A^P^, A^P^A^P^, A^P^A^P^A^P^A^P^ …, and

P = phospholipids and phosphonexuses

this models how a polypeptide (quantized in A), establishes direct relationships with a phosphomembrane, interdigitating with the surface phosphates. This clarifies the membrane protein folding problem [568]. Many hydrophobic and membrane-anchored macromolecules (e.g., channels [569], pores [570], basal body [571]) are positioned here [572].

*Non-ribosomal Peptides and Sulfated Compounds*. I can now fit nonribosomal peptides (NRPs, e.g., antibiotics, siderophores, cytostatics; [573])—secondary metabolites produced by a variety of microorganisms, many of which participate in intra- and intercellular signaling [574]. The translation apparatus does not generate NRPs but rather, it is thought, enzymes do [575]. Here, I model NRPs origins as

NRP ⇆ aa^x^ + Ⓡ

which fits onto [A^R^] ⇆ [A] + Ⓡ, where Ⓡ is the gyradaptive force (e.g., NTPs), aa is *any* amino acid [576,577], and ‘x’—given long-range thermodynamic interactions of greater exergy (G_XI–1_ and G_XIII_)—denotes any chemical modification or solution (H_2_O) with sufficient potential energy to facilitate NRP metabolism. The permutability of the gyrosystem, along with its adaptive capacity, reveals how antibiotics, over time, lose their efficacy and specificity [578]. [A] also positions phosphorus- and nitrogen-free molecules (C, H, O, and S) like sulfolipids [579] due to carbogyre attractorepulsion on the tertiary aminogyre. Additional evidence validating this thermodynamic carbyon-aminon relationship is found in ***aminocarbomatrices*** called peptidoglycans [580].

*CHNOPS*. How and why life is predominantly made up of hydrogen, oxygen, carbon, phosphorus, nitrogen, and sulfur is an unanswered question [581]. I show that the electrogyre (H, and all other elements), oxygyre (O), carbogyre (C), phosphogyre (P), ribogyre (N) and aminogyre (S) provide a coherent theoretical answer for how and why the living cell is 98% CHNOPS.

*From Protein to DNA.* The amino acid is the molecular building block for the polypeptides that exist in all life forms known to science. With the ribogyre and aminogyre, I have provided an axiomatically constrained and empirically consistent system for understanding the origin and evolution of these biomolecules. The aminogyre makes some very profound and testable predictions about the specificity of the genetic code and how proteins behave, lengthen and shorten, and fold and unfold in response to physical and biometabolic changes or changes in genetic information content of RNA.

When considering the next evolutionary bound towards the origin of life, my attention turned to one particular class of proteins. The enzyme RNR is a crucial protein in the evolution of life because it and it *alone* performs an essential biochemical reaction: RNR converts a ribonucleotide to a deoxyribonucleotide [582]. Without this reaction, DNA would not exist and the living cell as I know it would not emerge. Thus, the RNR protein family, along with a cadre of nucleoproteins, is part and parcel of the very existence of genes and genomes—a veritable molecular bridge between the RNA and DNA worlds [583,584,585].

### 3.7. DNA

DNA is arguably the molecular capstone in the evolution of life. In revealing the structure of DNA, the “secret of life [586],” Crick and Watson set the stage for a new generation of scientists to find that there was a seemingly endless quest towards unraveling a profound mystery enshrouding that secret [587]. In this subsection, I fit some of the most important facts related to DNA onto a gyrosystem called the ***genogyre***. The genogyre provides novel viewpoints on the origin and evolution of genes, genomes, and chromosomes. Because the aminogyre is nested within the genogyre, the Matrioshkagyre organization is a spatiotemporal heuristic for how proteins regulate DNA structure and function.

*Deoxynucleotides and DNA Cis-Acting Elements*. The ***primary genogyre*** (Figure 2g (*i*)), fits the evidence about deoxynucleotide origin and evolution, where

D^3A^ ⇆ D^2A^ + Ⓐ

D = deoxynucleotide triphosphates (dNTPs: dATP, dGTP, dCTP, dTTP)

diphosphates (dNDPs), and monophosphates (dNMPs)

double-stranded (ds) and single-stranded (ss) DNA

A = A´, nucleic acid sensors; A´´, modifiers; and A´´´, interactors (3.6); any other germane protein.

This quarternary complex of DNA/sensor/modifier/interactor (D^3A^; gyrapex) is predicted to be unstable, with any one of the three nucleoproteins disassembling and cycling through the gyrosystem. In this regard, this model fits the known evidence about RNR(s) as sensing and converting

NMP → dNMP and

NDP → dNDP [582]

nucleotide kinase(s) to convert

dNMP → dNDP [588] and

dNDP → dNTP [589]

and nucleotide-binding protein(s) to store, remodel, and regulate (3.6) dNTPs and DNA. Vectorially, modeling the interactor (A´´´) cycling,

D^A´A´´^ + A´´´ → D^A´A´´A´´´^

depicts ***genognosis***, the process by which the deoxynucleotide or DNA receives and interprets IEM from the gyradaptive, repulsive aminon, and

D^A´A´´A´´´^ → D^A´A´´^ + A´´´

depicts ***genomnemesis***, where the deoxynucleotide or DNA retains information. Cycling of any aminon models its co-adaptational relationships with a ternary complex. Given that D accounts for ssDNA and dsDNA and given the quantal depth of the aminon, A´´´ fits an extremely large yet bounded number of *trans*-acting DNA-binding proteins [590,591]. Thus, this simple model for protein-DNA learning and memory explains how a protein physically identifies and targets (learns and remembers) a very specific *cis*-acting sequence (e.g., promoters, enhancers, terminators [592]). Continuous macrocosmic genognosis and genomnemesis is consistent with bioinformatic evidence [593,594,595,596] showing that *cis*-acting elements and motifs change in the evolutionary tree branches of life.

*Chromosomes and Chromatin*. The packaging of DNA by proteins occurs in all kingdoms of life. The ***secondary genogyre*** (Figure 2g (*ii*)) can be written as

D^3A^ ⇆ [D^A^] + 2Ⓐ

Here, the gyrobasal [D^A^] represents what is theoretically called a ***genonexus***. A genonexus is a length of DNA (gyromodule) gyrolinked (G_X_) by proteins (aminons that *are* aminonexuses). In this scenario, the gyrolink is A´´´, which models nucleoid proteins [597] in eubacteria and chromatin proteins (especially histones [598]) in archaebacteria and eukaryotes. In eukaryotes in particular, D^A^ is a mononucleosome, 146 base pairs of DNA spiralling around an octamer of histones H2A, H2B, H3, and H4; (Figure 4g; [218]), D^A^D^A^ is a dinucleosome [599], and D^A^D^A^D^A^D^A^D^A^…; any longer genonexus models “beads on a string [600].”

Although I modeled the diaminon that is the thermodynamic driving force for genonexus formation as **A**´ and **A**´´ (2Ⓐ = **A**´**A**´´), the secondary genogyre fits any gyradaptive diaminon. For this schema, genonexus packaging and organization is modeled vectorially:

D^A´A´´A´´´^ → [D^A´´´^] + 2Ⓐ

and breakage, remodeling, and mobilization, is depicted as:

[D^A´´´^] + 2Ⓐ → D^A´A´´A´´´^

Elaborating from above, **A´´´**, models *trans*-acting factors, specifically activators [601,602], repressors [603], chromatin remodeling factors [604], among others. **A**´´ represents the full complement of DNA-modifying enzymes (3.6), all of which are demonstrably participants in the generation and organization of the genonexus. Indeed, DNA repair [605], recombination [606], transposition [607,608], and sequence rearrangements [609] are all modeled onto the secondary genogyre as the gyradaptive effects of the diaminon singularity. Shorthand, this would be:

chromatin ⇆ nucleosomal DNA + protein sensors/modifiers

*DNA Structure*. DNA is a double helix of antiparallel dNMP single strand polymers (a chemically modified ribonexus called a ***deoxyribonexus***; not to be confused with the genonexus, which is the deoxyribonexus dynamically gyrating around aminonexuses). Note that the molecular genetical homeostasis of the antiparallel deoxyribonexuses orbiting the aminon singularity is comparable to the biophysical homeostasis of the antiparallel phosphonexuses orbiting the carbyon singularity (3.4). Although left-handed A-form DNA can be engineered, only right-handed B- and Z-form DNA occur naturally [610,611]. DNA is exclusively right-handed helix in life—because the aminogyre is right-handed (G_XII_). Moreover, DNA wraps around the histone octamer in *only* a left-handed fashion due to oscillating chiralities during gyrosystem complexification (G_XII–3_; Figure 4g). Keeping with the theoretical vernacular, then, the genonexus is visibly a ***genohelix* [612]**. As the genohelix gyrates upon itself, it forms more and more complex ***genomatrices****,* the higher order structures known as the 30 nm solenoid [613], chromatin loops and fibers [614], and mitotic chromosomes [615].

*DNA Virus*. Given that RNA is the evolutionary predecessor of DNA, a common theme in evolutionary virology is that DNA viruses are evolved from RNA viruses [616]. Being that the deoxyviral particle is inert without the living, dividing cell, the second genogyre models the generation of the viral genonexus:

Viral genome/3A ⇆ Viral genome/packaging proteins + 2A

This simple model thus portrays the origin and evolution of all DNA viruses; the logical neologism for such a quantized DNA-protein (or RNA-protein) complex in the gyrobase is a “viron”. In this regard, the large-scale cycling and metabolism of virons through genomes and cycling of genomes throughout the biosphere is modeled by a ***macrogenogyre***. The genogyre and ribogyre demonstrate that DNA and RNA viruses, respectively, emerge prior to the dividing cell and are a natural consequence of universal expansion towards the origin of life.

*Origin of DNA Content, Mutations, and Other Problems*. How new open reading frames—regions of the genome that are complementary to RNA sequences—come into existence is a core problem of evolutionary biology and the subject of intense phylogenetic and bioinformatic study [617,618]. One favored explanation is Ohno’s gene duplication model [619,620]. Still, in the spirit of scientific transparency and honesty, the duplication idea does not address how the *first* or *novel* genes emerge. To address this dilemma, I applied the genogyre accordingly. In the ***tertiary genogyre*** (Figure 2g (*iii*)), the gyrobasal [D] loses support of the triaminon, and takes on direct thermodynamic relationships with the ribon (G_XI_). This ribon-deoxyon interaction models how ribons or ribonexuses exert the attractorepulsive force on the ***genons*** (the quantal/particulate model of the genogyre; Figure 2g (*iv*)), literally “linking” them together:

[D] = D_R_, D_R_D_R_, D_R_D_R_D_R_, D_R_D_R_D_R_D_R_…

where R = individual NTPs, exons, introns, and regulatory RNAs. In turn, D models the corresponding dNTPs and DNA sequences. This theoretical relationship thus flips the conventional view on its head: RNA is the unstable, ever-changing template upon which the gene and genome forms, expands, and adapts. Compressing the secondary and tertiary genogyres, I have

[D^A´´´^] → [D] + Ⓐ

a 2°/3° alternagyre that shows how the aminon singularity attracts and unfolds the gyrapical genonexus (DNA-protein), thereby permitting interface with the RNA template. The opposing directionality

[D] + Ⓐ → [D^A´´´^]

shows how the aminon repels the gyrobasal genonexus (DNA-RNA) back to its high energy state, evicting the RNA. A full cycle, a ***genocycle***, permits the adaptation of genic and genomic (coding and non-coding) sequences. In other words, RNA-directed changes to the DNA sequence undergo proteinaceous genognosis (where [D^A´´´^] is the gyrapex, the learning gyrostate of this alternagyre) and ultimately are genomnemonically stored (where [D^A´´´^] is the gyrobase, the memory gyrostate of the secondary genogyre). The theoretically-defined mnemonic character of DNA-protein is more stable than that of DNA-RNA, as the exergy and attractorepulsive effects of the aminogyre are relativistically less those of than the ribogyre (see G_XIII_).

This model (also see Figure 3h) has the potential to facilitate understanding of a variety of molecular genetic problems. For example, it resolves the origin and evolution of genes and gene families [621,622], origin of intronic sequence in RNA and DNA [623,624], directed mutation controversy [625,626,627], why 80-90% of a genome has transcriptional output [628], transcription-associated recombination [629], how RNA mediates epigenetic reprogramming of DNA [630], RNA-templated DNA repair [631], site-specific changes in viral genomes [632,633], and, since RNA harbors the genetic information memory for templating DNA, how lateral gene transfer is widespread between, among, and within different genomes and differing organisms [634,635,636].

*dNTP Pools*. Regulating the levels of dNTP pools is fundamental for proper cell function [637]. Moreover, DNA replication—which is necessary for fidelitous cell division—is preceeded by a wave of dNTP accumulation [638] that is tightly regulated [638,639,640]. In the tertiary genogyre, [D] also models the thermodynamic relationship between the stoichiometric levels and pools of NTPs (ribogyre) and dNTPs (genogyre) unincorporated in RNA and DNA, respectively. This theoretically defined dNTP-NTP feedback jives with the empirical necessity of the chemical energy from ribonucleotides to drive biosynthesis and transport of sugars, membranes, and organelles (3.6) that concresce as new cell material. In the 2°/3° alternagyre, the A that cycles corresponds to RNRs—sensing levels of dNTPs and NTPs—and other protein sensors such as components of the DNA damage machinery that work during cell cycle checkpoints [641,642].

**Figure 5:**
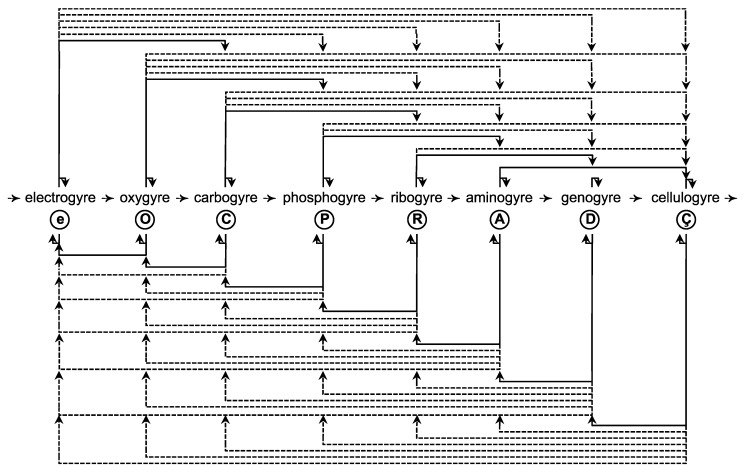
Left-to-right theoretical framework. The arrowheads between the gyrosystems (center flow line) represent both the evolutionary process leading up to the origin and evolution of cells and how existing cells work. The self-directed arrows that are above and below the gyrosystems represent autoregulation. The arrowed lines above the center line depict the feedforward between and among gyrosystems; those below the line depict feedback. The gyrosystem interactions discussed the most in the text are labeled as dark lines. The dotted lines represent empirically definable or predicted gyrosystem flow. Those arrowheads that flow into the electrogyre (the photon from the left) and flow out of the cellulogyre (to the right) depict the evolutionary steps prior to and following the origin of visible matter and the cell, respectively; these are either briefly mentioned or not discussed in this study. Please note the unity of reality and life as revealed by this theory.

*Flow of Molecular Genetic Information*. The current idea for how genetic information flows in cells involves only three components: DNA, RNA, and protein. While this reductionist idea has been powerful, there have been calls of a conceptual crisis [643,644] and for shifts to systems thinking [645]. In other words, these critics imply that understanding the flow of genetic information requires understanding more than just genetic information. As modeled by this interdigitated theoretical framework (Figure 5), genetic information flows coherently from biochemical and biophysical IEM:

→ electron → water → organic matter → phosphochemistry and membranes

→ RNA → protein → DNA → cell

The relationships of gyrosystems—as shown in the flow diagram—solves many of the unsolved questions in molecular biology [646]. Moreover, the flow diagram provides an alternative perspective to the central dogma, Mendelian genetics [647,648], neo-Darwinian selection of random mutations [649], and selfish gene theory [650] on matters such as genomic stability [651,652], adaptability [653], and inheritance [654].

*From DNA to the Living Cell.* I have fit the modern evidence related DNA to the genogyre and have theoretically confirmed the nature and composition of the DNA world that existed in evolution of life on Earth [584]. With the genogyre, I have a system of unreplicated DNA within a phosphomembranous sac; in other words, the genogyre does not explain life as I know it. Because a correct theory of life must explain not just how but *why* a living cell divides, I now turn to DNA replication and cell division.

### 3.8. Cell

A vast expanse of the scientific firmament implicitly accepts two core premises of cell theory [655]: (*i*) the cell is the basic unit of life; and (*ii*) all cells emerge from other cells by division. While the first premise of cell theory is definitionally confining, it is not theoretically problematical. The second premise, however, is deeply troubling, because it implies that the origin of first cell is impossible to explain by *reductio ad absurdum*. In considering the origin of life, I have already accounted for and modeled empirical evidence related to the physical, chemical, biochemical, and molecular systems upon which a living cell relies (3.1–3.7). While life can and does exist in a quiescent state for an extended period of time as a spore [656] or in a terminally differentiated state, these cellular forms emerge only from a cell that has potential to divide. Thus, in order to model the origin of life, I had to model the origin of the first cell division. The ***cellulogyre*** (Figure 2h) is a gyrosystem that reveals that the first cell division on Earth was executed in a fashion identical to a dividing cell that an investigator examines in this very epoch of basic and clinical research.

*DNA Replication and Cell Division*. During the genesis, repair, and remodeling of genome sequence and order, the pool of unincorporated dNTPs expands within the gyrobase of the tertiary genogyre (3.7). However, as the other gyrosystems that are subsumed by the genogyre are likewise expanding, and the genogyre is the end point of IEM deposition, the genogyre shunts IEM back within itself—one of the most prominent examples of this is phosphogyre expansion, as new membrane synthesis and expansion occurs during the process leading up to cell division [657,658]. In both the ***hapcellulogyre***(Figure 2h (*i*); 1N, one set of homologous chromosomes (haploid)) and ***dipcellulogyre***(Figure 2h (*ii*); 2N, two sets of chromosomes (diploid)), respectively, DNA replication [659,660] is modeled as repulsion by the (di)genon, the thermodynamic singularity within the cell (Figure 2g (*iv*) and 2h):

[Ç^D^] + Ⓓ→ Ç^2D^

[Ç^2D^] + 2Ⓓ → Ç^4D^

and cell division and nucleoid or chromosome segregation [661,662], by its attractive force:

Ç^2D^ → [Ç^D^] + Ⓓ

Ç^4D^ → [Ç^2D^] + 2Ⓓ

Quite parsimoniously, the hapcellulogyre models the replicated DNA state (Ç^2D^; gyrapex) and the pre-replicated state (Ç^D^; gyrapex) in archaebacteria [663], eubacteria [664,665], plastids and mitochondria [666,667], and haploid eukaryotes. The gyradaptor, Ⓓ, represents the G_0_ state [668]—the non-replicative, quiescent, membrane-encapsulated genon—of the haploid cell cycle (see below). The dipcellulogyre models the replicated (Ç^4D^; gyrapex) and pre-replicated (Ç^2D^; gyrobase) states in multicellular eukaryotes that are not metazoan [669]; the gyradaptor, 2Ⓓ, represents the diploid cell cycle G_0_. In this regard, two nonlinear steps explain the origin of the eukaryotic cell: (*i*) expansion of subsumed gyrosystems on the digenon (2D) on the hapcellulogyre to a point of critical IEM content elicits tetragenon (4D) formation; (*ii*) gravitational collapse (attraction by the genon and all inherent gyrosystems) from within the dipcellulogyre shunts the IEM into gyrosystems and complexifies from within to without (endomembrane system (3.4); novel RNA, protein, and DNA architecture (3.5–3.7); mitosomes, hydrogenosomes, and plastids [670,671])—and a hapcellulogyre emerges nested like a Matrioshka doll within a dipcellulogyre. This is consistent with autogenic hypotheses [672] yet provides an alternate view to mainstream serial endosymbiotic theory [673,674] and the hydrogen hypothesis [675].

The ***acellulogyre*** (Figure 2h (*iii*)), the gyrobasal [Ç] models an achromosomal, anucleate, or DNA-lacking cell or cell system, e.g. aneuploidy [676], ρ^0^ mitochondria [677], hydrogenosomes, platelets [678], red blood cell [679], or apoptotic or differentiating cell [680,681]:

Ç^2D^ → 2Ⓓ + [Ç]

Ç^4D^ → 4Ⓓ + [Ç]

*Cell cycle*. The four-dimensional cellulogyre is another way of understanding the three-dimensional cell cycle (***cellulocycle***): G_0_ is the singularity; Restriction point, or start [682], is the gyrobase; G_1_ phase [683], is the transition from gyrobase to hemi-bas-apex (HBA, halfway from the ground to excited state); S phase, or DNA replication [684], is the transition from HBA to gyrapex (the excited state, Ç^4D^ and Ç^2D^); G_2_ phase [685] is the transition from gyrapex to hemi-apica-basal (HAB, halfway from the excited to ground state); M phase [686] and cytokinesis, or cell division [687], is the transition from HAB to the gyrobase (the ground state, Ç^2D^ and Ç^D^). The more proximal the ***cellulon*** (Figure 2h (*iv*)) particle is to the genon singularity, the more unstable and rapid the interconversion of these phases, *i.e*., the oscillation between two extreme gyrostates. This rapid oscillation explains why, for example, the cell cycle in the *Drosophila* syncytial blastoderm has no gap phases [688,689].

From an evolutionary standpoint, the earliest and most basic cells (archaebacteria, eubacteria) are closest to the genon and thus thermodynamically unstable, with fast division times (20–30 minutes). The more evolutionary recent cells, eukaryotes, are furthest from the genon singularity and take longer to complete their cycles around it (yeasts, ~1.5 h, protozoans, 6–8 h, somatic cells 10–24 h). Thus, the cellulogyre models the cyclical nature of the living cell systems and the evolution of these cycles in said systems [690,691].

*Cellular Form*. One of Haeckel’s enduring naturalistic offerings is a magnificent late 19^th^ century view of the numerous forms of life [692]. With the advent of deconvolution, confocal, and two-photon microscopy [693,694], scientists have obtained a rich and detailed catalog of cell form and function to supplement Haeckel’s vitascape. Despite this supreme microvision, one cell biologist publicly lamented about the limits of the trade [695]. To salve this concern and to understand the variety of cell forms and functions, I applied the cellulogyre accordingly. For example, the gyrobasal [Ç^D^] represents a haploid cell polymer, a ***hapcellulonexus***,

[Ç^D^] = Ç^D^, Ç^D^Ç^D^, Ç^D^Ç^D^Ç^D^Ç^D^…

and the gyrobasal [Ç^2D^] represents a diploid cell polymer, a ***dipcellulonexus***,

[Ç^2D^] = Ç^2D^, Ç^2D^Ç^2D^, Ç^2D^Ç^2D^Ç^2D^Ç^2D^…

Hapcellulonexuses and dipcellulonexuses are predicted to be the most basic higher-order organization of the cellulogyre. These nexuses aggregate and fractalize into *hap-* and ***dipcellulomatrices***. These single cell layer matrices or surfaces are manifest, for example, in leaves [696], microbial mats [697], fruiting bodies [698], or a mere blade of grass. These matrices assemble into higher-order structures as well, as several haploid and diploid algal, fungal, and plant cells cell systems grow and/or exist in gyratory form (***cellulohelices***; Figure 4h). The oocyte and many cell types are ***cellulospheres***, having the hallmark spherical form that is found throughout nature and a consequence of omnidirectional expansion of the genogyre within the omnidirectionally expanding cellulogyre. Paleobiologically, the acellulogyre models the evolutionary remains of less complex or thermodynamically unstable cellular forms: plant fossils, stromatolites, and fossilized microbial mats [699]. In this regard, acellulons contain the membrane signature of life and the protein networks that hold them together [700] but are predicted to lack their genomes.

The core features of the gyromodel resolve the matter of how cellular shape and size is established and maintained [701,702]. For instance, the relativistically high energy, unstable, excited state of the cell (Ç^2D^ and Ç^4D^) is modeled as cell learning, or ***cellulognosis***. The lower energy, stable, ground state (Ç^D^ and Ç^2D^, respectively) is modeled as ***cellulomnemesis***. Theory thus demonstrates that the cell retains all of its evolutionary history embedded within its biomolecules and particles. Precisely mirroring this theory, the modern cell maintains its overall spatiotemporal information by adapting to acute and/or chronic physical, bioenergetic, and pharmacological cues [703,704,705] that are received, dissipated, and homeostatically integrated in gyrosystems *within* itself. Other features of the modern cell are noteworthy [706,707].

*Aging and Death*. Because cell death is modeled by the acellulogyre, aging and death—another unclear and unsolved problem of biological science [708,709,710]—is clarified. As modeled in this theory of life, a cell divides as long as IEMs flow through and from the subsumed gyrosystems (from the electrogyre to the cellulogyre). In this regard, since the cell is composed of CHNOPS and other chemical elements that ultimately emerge from and is modeled by the electrogyre, this matter gyrates on a grand scale from the electrogyre to the cellulogyre and from the cellulogyre back to the electrogyre. Senescence—the process of cellular deterioration—is modeled as the thermodynamic instability of the cell or cell system due to the gyrosystemic attractive force increasingly countervailing but not surmounting the repulsive force over evolutionary spacetime. Death is a consequence of gravitational collapse of the cellulogyre into its singularity (genon) due to unobstructed attractive force exerted by, on, and within all subsumed gyrosystems. Consistent with this explanation, in death, the undividing cell begins to break down and, without preservation, ultimately recycles its chemical molecules through the biosphere. The biotic death spiral [711] is more than just a play on words; it reflects the unavoidable gravitational collapse of the cellulogyre.

*Meiosis and sex*. The origin of sex is a nebulous affair, having occurred in the surreptitious record of life’s evolution. While there are many ideas related to sexual origins (for example, [712,713,714]), a common theme is the oscillatory diploid-haploid life cycle between gamete fusion (syngamy) and meiosis [715]. Recall that, in a gyre, a mIEM particle can exist in only one of the three spatiotemporal locations: the excited state, the ground state, or in the singularity itself (2.3.3). With this in mind, the attractive force of the genon on the dipcellulogyre models oogenesis [716,717],

♀: (*i*) Ç^4D^→ (*ii*) Ç^2D^ + 2Ⓓ → (*iii*) (Ç^D^ + Ⓓ) + Ç^2D^ → (*iv*) Ç^D^ + Ⓓ + Ⓓ + Ⓓ

(*i*) the attractive genon induces the first meiotic division of the primary oocyte (Ç^4D^); (*ii*) this produces the secondary oocyte (Ç^2D^) and the first polar body (2Ⓓ); (*iii*) the secondary oocyte undergoes the second meiotic division (substituting the hapcellulogyre here), yielding the mature ovum and a polar body (Ⓓ) and the first polar body expands (Ç^2D^); and, (*iv*) being that the dipcellulogyre gyrobase is the same as the gyrapex of the hapcellulogyre (*cf*. Figure 2h (*i*) and (*ii*)), both particles gravitationally return to the genonic state (Ⓓ + Ⓓ). Modeling spermatogenesis, I have,

♂: (*i*) Ç^4D^→ (*ii*) 2Ç^2D^→ (*iii*) 4Ⓓ

This flow diagram, in parsed fashion, shows: (*i*) the primary spermatocyte (Ç^4D^) undergoes mitotic division (dipcellulogyre); (*ii*) both cells emerge from the division as secondary spermatocytes (2Ç^2D^; one is Ç^2D^, other is rapidly 2Ⓓ → Ç^2D^); (*iii*) these cells divide, then arrest in G_0_, the hapcellulogyre singularity (2Ⓓ + 2Ⓓ = 4Ⓓ).

So, in the end of gametogenesis, the sperm (Ⓓ) is one mating type or gamete and the egg (Ç^D^) is the other gamete. Modeling sex, the hap- and dipcellulogyres *themselves* model the manner by which the sperm (genon singularity) fertilizes (exerts a repulsive force) on the egg (gyrobasal cellulon), forming the zygote (lifting it to the high energy state), which ultimately is repelled by DNA replication (digenon expansion) to the highest potential energy state (Ç^4D^). Oversimplifying:

Ç^D^ + Ⓓ → Ç^2D^ + 2Ⓓ → Ç^4D^.

Summarizing, a single genonic quantum can exert *either* the attractive force (meiosis) *or* the repulsive force (sex), but cannot execute both phenomena simultaneously. Note the oscillation between countervailing forces is a corollary to gyraxioms that treat other oscillating features of the gyromodel (G_V_ and G_VI_). Importantly, this theory conforms well to the hormonal cycles that drive gametogenesis and the juxtaposition of gametes [718,719,720,721,722,723]. The genon-as-singularity concept is presented in Figure 3 (i). Understanding the large-scale relationships of hormones within the diploid-haploid life cycle can be considered as Matrioshkagyres (Figure 3e,j). Finally, the cellulogyre and intrinsic gyrosystems are a concrete substitute to Weismannian thinking [724].

*C-value enigma*. The C-value enigma states that less evolutionary developed cell types have greater genome size than more complex cell systems [725]. Solving this enigma requires reviewing the evolutionary trajectory just prior to the emergence of the cellulogyre: the genogyre is the final point of deposition for all of the accumulated IEM subsumed within itself (Figure 5). A consequence of incorporating all of this information, energy, and matter is genogyre expansion, which models genomic expansion (complexification). The closer the genon is to the aminon singularity, the more unstable and simple the genome; the further from the singularity, the greater the complexity. On a macrocosmic (*i.e*., kingdom-wide, organism-wide) scale, the cellulon expands (complexifies) to accommodate genon expansion within itself.

By way of reminder, just as each particle in a gyrosystem oscillates between unstable and stable states, so too the gyrosystem itself (which should be thought of as a quantum or as one or more particles (G_I_, G_III_) oscillates between unstable and stable states within another gyrosystem. Whereas the former oscillation is spatiotemporally brief, the latter is more prolonged, as there is more IEM to mobilize. So, then, in complexifying, the cellulon becomes the nascent point of IEM deposition in lieu of the genon. The closer the cellulon particle is to the genon, the more complex the genome and less complex the cell system; the further the cellulon is from the genon, the more complex the cell system and less complex the genome. This dynamic cellulon-genon relationship affords a novel perspective on the C-value enigma that echoes a prior hypothetical solution [726].

*Extracellular Material and Integument*. Without thermodynamic support of the genon, the cellulon has direct relationships with the aminon and subgyres (G_XI_, G_XI–1_). Briefly, this thermodynamic shunt helps clarify not only the proteinaceous extracellular matrix that “links” cells together [727,728],

[Ç] = Ç_A_, Ç_A_Ç_A_, Ç_A_Ç_A_Ç_A_Ç_A_….

but also the integumentary system (e.g., skin, feathers, beaks, scales, hair, shells, hooves, tusks, and claws; [729,730]). The asymmetric chirality of these extracellular structures appear as whorls, helices, or spirals [731,732] and are due to the gyradaptive forces within the cell.

*Circadian rhythms*. A large number of cells have an internal “clock,” a system that is responsible for maintaining periodic oscillations between states of metabolic, physical, and chemosensory activity and inactivity [733,734]. These temporal rhythms are called circadian because they occur over a 24-hour period. Although there is compelling evidence that changes in RNA and protein expression levels are associated with changes in these rhythms [735,736,737] and there are models to make sense of this data [738], there is no unifying theoretical framework.

Applying the ohiogyre frame here helps clarify these rhythms. Recall that a lunar core (an macroxyon with a macroelectron singularity) gyrates around a planetary core (a macroelectron with macrophoton singularity) in an ohiogyre (3.2). Under this scheme, the cell (a cellulon with a genon singularity) is modeled as rotating around a genomic singularity (a genon with an aminon singularity) in an ohiogyre. That is to say, the oscillating cellular particle (one cell in a cell population) orbits around the attractorepulsive genon singularity (quiescent, unreplicating, or post-replicative cells in that population; [739]). Because one cell cannot be understood apart from its cellular heritage and the cell is dependent upon all of the internal and external thermodynamic cues (e.g., ions, nutrients, energy; G_IV_, G_VIII_), circadian rhythms may be considered only from the history of the cells under examination.

*The Self-organization and Self-regulation of Life.* This theory concomitantly treats both the microevolution and macroevolution of life. From a microevolutionary standpoint, the gradual changes in individual cell are modeled by the countervailing adaptive forces—attractorepulsion, creatodestruction, expansocontraction—that emerge from within the cellulogyre and can be experimentally observed (e.g., [740]). Moreover, the gyromodel is in lockstep with mainstream thinking regarding the self-organizational properties of cells [741]. On a macroevolutionary scale, the expansion of the universe exerts a thermodynamic repulsive force through the macrogenon with ultimate deposition into the ***macrocellulogyre***; this provides a simple explanation for microbial ontogenesis, phylogenesis, and evolution [742]. Since one cell consumes, retains, and/or expels parts or whole of another cell during phagocytosis [743], endocytosis [744], and endosymbiosis, cell mass is modeled as cycling through a macrocellulogyre.

In conclusion, the cellulogyre demonstrates that while the Latin phrase *Omne vivum ex vivo* (“all life [is] from life”) is true, it is ontically incomplete. What cell theory was unable to explain—how the first cell originated—this theory explains as the turning and churning of information, energy, and matter in a widening gyre.

## 4. Conclusions

**Figure 6:**
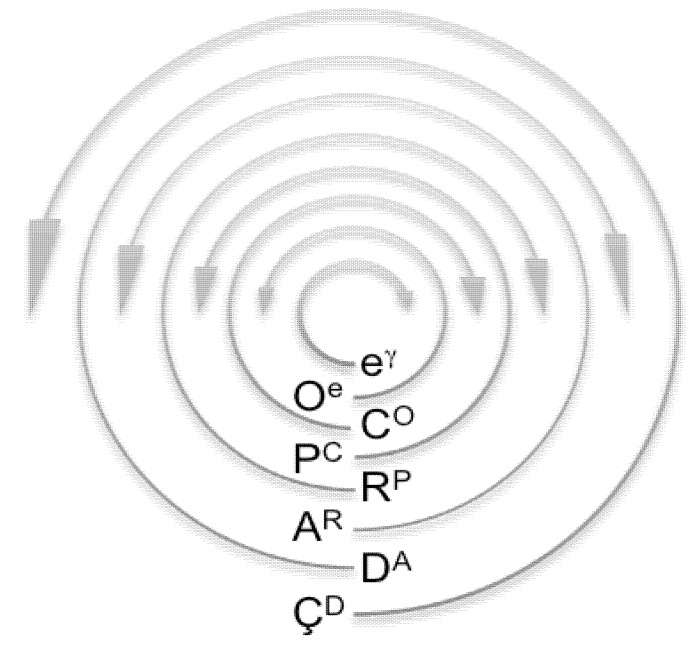
Within-to-without theoretical framework. The electrogyre (where e^γ^ denotes all lepton potentialities) is within the oxygyre (where O^e^ denotes all oxyon potentialities) which is within the carbogyre (where C^O^ denotes all carbyon potentialities) which is within the phosphogyre (where P^C^ denotes all phosphon potentialities) which is within the ribogyre (where R^P^ denotes all ribon potentialities) which is within the aminogyre (where A^R^ denotes all aminon potentialities) which is within the genogyre (where D^A^ denotes all genon potentialities) which is within the cellulogyre (where Ç^D^ denotes all cellulon potentialities). Matrioshkagyres—nested antichiral gyres—achieve homeostasis by reducing the rate of IEM metabolism and flow between, among, and within gyrosystems. Time flows from within to without: microcosmically, the rate of each cycle decelerates, as an electron cycles much faster than a cell cycles; macrocosmically, the rate of each cycle decelerates, as planetary axial rotation cycle is relativistically faster than the existential cycle of a particular cellular species. Please consider the widening gyre in light of universal expansion.

I have compiled and unveiled an axiomatic, experimentally testable, empirically consistent, heuristic, and unified theory of life. Given the breadth and depth of this work, I summarize the theoretical organization in two different ways. The first (Figure 5) is a left-to-right schematic that accounts for feedforward and feedback between, among, and within gyrosystems. The second (Figure 6) is a within-to-without schematic that reveals gyre nesting and chiral toggling. These two schemes afford complementary perspectives on how cellular life originates, evolves, exists, and functions.

### 4.1. Theoretical Solutions, Limitations, and Expectations

Although there have been a handful of theories that model the cell and the origin of life [745,746,747,748,749,750], to the best of my knowledge, I am presenting the first and only scientific theory of life from the quantum to the living cell. On this basis, my theory is *sui generis*. I broach the correctness of the theory, reiterate a handful of original solutions to protracted scientific problems, and discuss several issues related to comprehensiveness. I pepper this subsection with several theoretical predictions.

*Solutions*. A correct theory should not only explain *how* things work but explain *why* things are the way they are. This theory of life is correct—in precise accordance with natural laws and scientific truths. The ergodic gyromodeling of the origin of both the living cell and the biosphere is correct. The evolutionary positioning of celestial bodies, chemical elements, biogeochemical cycles, biomolecules, and genetic material is likewise correct.

I refer the reader to the Theory section for a complete presentation of theoretical answers to many of science’s most challenging questions [751,752]. Here, for brevity, I highlight only one theoretical solution for each gyrosystem. The electrogyre explains quantum gravity, unifying quantum mechanics and general relativity in a frame beyond the standard model [753]; the oxygyre explains the mysterious properties of water [133]; the carbogyre explains the emergence of hydrocarbons in the Earth’s mantle and crust, resolving the biotic/abiotic petroleum debate [754]; the phosphogyre explains why phosphorus is “life’s bottleneck [755]” and the dominant roles of phosphate in biology [756]; the ribogyre solves the problem of novel genetic information [757,758,759]; the aminogyre explains the origin and nature of the translation apparatus, one of theoretical biology’s grand unsolved problems [488,760]; the genogyre clarifies the correct relationship of DNA, protein, and RNA, quelling anonymous protestations against the central dogma [761,762]; and the cellulogyre reveals that life originates in any biosphere wherever the thermodynamics of information, energy, and matter are accommodating, consistent with ideas regarding hierarchical complexification of and in the universe [763]. Together, the theoretical framework confirms what many modern theoretical physicists have proposed: that the classical world is “quantum all the way [764].” The gravity and implications of these solutions are discussed below.

*Comprehensiveness*. Although I fit the fundamental, structural data from physics, chemistry, and biology, as they are broadly defined, I did not incorporate *all* of the evidence in those fields. Moreover, I addressed major scientific enigmas, anomalies, and paradoxes but did not address minor puzzles and curiosities. Based upon the successful record of fitting data to the eight presented gyrosystems, I expect more comprehensive data fitting to be profitable and of general interest.

*Scope*. The theory outlined in this manuscript is limited in scope. I did not provide gyrosystems to model much of the scientific evidence related to astrophysics, particle physics, and cosmology before the electrogyre, nor did I integrate organismal, ecological, and ethological data after the cellulogyre. I predict that further gyromodel application will reveal its explanatory breadth and power. For example, given that complexity theorists find there to be a unifying organization in ecosystems, language, and economics [765,766,767], I predict the gyromodel will find applications in these subject matters. I also expect the gyromodel to fit data related to the ontogenesis and phylogenesis of *Homo sapiens*.

### 4.2. Laws of Nature

This theory demonstrates that the complex biology of life obeys known natural laws as they pertain to physics and chemistry. As broadly defined, a law of nature is a physical or scientific principle that is a systematic, general, and formal statement derived from empirical observations of natural phenomena [768,769]. Natural laws have several properties: basic—an essential, necessary, and common foundational principle; universal—apply throughout the visible universe; true, or unfalsifiable—no evidence to contradict its validity; absolute—not subject to conditions or limitations; immutable—unchanging, stable. With this work, I have given incontrovertible proof for the following eight laws of nature, most of which have already been articulated and promoted.

*Fourth Law of Thermodynamics*. The theoretical framework sheds light on how life maintains order and complexifies in spite of entropy: the repulsive force of the gyradaptive singularity elevates a particle to its excited state, offsetting the effects of it cycling to the ground state. The gyromodel thus confirms the existence of the fourth law of thermodynamics [770], the ordering law of the universe.

*Law of Polymers*. A key theoretical implication is that the polymeric form is compulsory in all IEM arrangements—as modeled by the secondary and tertiary gyrobases. This is not surprising, as a every known living system requires biomolecular oligomers of a certain length [771]. Thus, the gyromodel reveals a natural law related to IEM organization: a law of polymers.

*Law of Vortex Motion*. This theory conclusively demonstrates that all physical systems, particles, and phenomena in the microcosmic and macrocosmic realms obey a vortical trajectory. In so doing, the framework validates the Democritean assertion in the Model section: vortex motion is a natural law.

*Law of Correspondence*. The theoretical framework shows that biopoiesis—the evolution and origin of life—is recapitulated in any and every extant cell. Furthermore, the electrogyre—in accurately depicting quantum gravity—shows there to be one model that explains the structure, function, and character of both cosmic and atomic phenomena. Hence, in proving correspondence between the macrocosmic and microcosmic realms through all gyrosystems, the Hermetic and Bohrian principle of correspondence [772] is elevated from a philosophical and quantum mechanical principle to a natural law.

*Law of Complementarity*. Bohr also proposed a complementarity principle—*i.e*., objects have multiple contradictory properties—to describe, for instance, the wave-particle duality of the quantum. The gyromodel and its axioms elevate this principle to a natural law by proving the complementary necessity of attraction and repulsion, anabolism and catabolism, learning and memory, spacetime position and trajectory, excited and ground states, solids and gases, units and polymers, among other pairs of phenomena.

*Law of Relativity*. Because the complementary pairs of phenomena, IEM, and states are *always* relative to any quantum, particle, or gyre of the gyromodel—and hence ever changing—this demonstrates that relativity is not only theory but a law of nature.

*Law of Trimergence*. Every majorgyre of the theoretical framework emerges with, cycles as, and is sustained and unified by a quantal triad. The triune organization of nature is observable in, for example, the three generations of leptons, three oxygen atoms in carbonic acid, the three phosphates in NTP, three RNAs in protein synthesis, three elemental forms (metals, metalloids, non-metals), three phases of water, triplet genetic code, and IEM. This tri-emergent phenomenon (***trimergence***) is thus a natural law.

*Law of Unity*. Each gyrosystem defies simplification; experimental analysis of a gyrosystem in purported isolation indubitably fails to account for sub- or supervenient systems, particles, and processes (Figure 5). The complexity of life thus surreptitiously withheld a law of unity that had been intimated [773,774]. In light of theory and this natural law, although paradoxically reducible to its component parts, a cell is rightfully indivisible, a unity with and in the evolving universe. This natural law decrees that physical reality is one.

A foundational goal of science is to identify and understand the physical laws that govern the visible universe [775,776]. The demonstration of new laws of nature is thus an important scientific achievement.

### 4.3. Theoretical Proofs and Implications

I have arrived at several compelling proofs from this theory of life; on this matter, proof is defined as extraordinary evidence that establishes a fact or the truth of a statement. In this section, I detail theoretical proofs related to origins, time, order, adaptation, evolutionary emergence, and life on Earth and in the universe. I conclude this subsection with one point regarding metaphysics, another regarding causality and necessity, and another on the relationship of this theory to the epistemological progress of science.

*Origins*. In this theoretical study, I have demonstrated that each gyrosystem singularity represents the origin of that gyrosystem. In other words, the singularity is the beginning and the end, the thermodynamic source and the sink of each cycle of IEM through a gyre. Because the dwell time in each singularity is immeasurable—as each singularity is potentially infinite—it has been an empirical challenge to recognize its existence without this theory. The theoretical model harmonizes with Cantor set theory [777,778] by demonstrating that the origin of life is a consequence of iterative nested origins or singularities (Figure 3e,i, and Figure 6), which, despite relying on the same core model, are paradoxically more than one *kind* of infinity.

*Arrow of Time*. Eddington equated entropy with “the arrow of time” because of time’s asymmetry in observable physical processes and in evolution [779]. Theory accurately depicts the vectorial nature of time as IEM flow from within the electron to the cell as nested sets of singularities (Figure 6). Macrocosmically, time progresses from Earth’s origin (4.6 billion years ago) to the origin of the first living cell (~3.4 bya, the fossil record of cellular life [780]). Microcosmically, from electron cycling to cell division, time decelerates from ~10^−8^ seconds to 90 minutes (doubling of typical yeast cell).

*Order and Disorder*. Given the law of relativity, IEM order and disorder are demonstrated to be relative to the singularity. Further, given the law of complementarity, universal order and disorder paradoxically co-exist. In proving this contradictory fact, my theory does not “collapse in deepest humiliation [781],” but rather reflects and honors the *true* nature of the physical world.

*Adaptation*. This theory explains that all adaptation is the emergent, cumulative, and ongoing learning and memory of oscillating gyrosystem particles due to the attractorepulsive, expansocontractive, and/or creatodestructive force(s) of the quantal singularity. Because every gyrosystem adapts, this explains how and why physical, chemical, molecular, and cellular systems always seek homeostasis [782,783].

*Evolutionary emergence*. The Darwinian theory of evolution by natural selection does not address how novelty emerges in the universe [784,785]. As modeled here and as observable in the natural world, gyres expand and develop as a consequence of continual IEM flow, rearrangement, and coalescence. When the maximum carrying capacity of a gyrosystem is reached, it extrudes IEM due to spatiotemporal constraints imparted by sub- and supragyrosystems; this models self-organized criticality [786]. Gyrosystem collapse thus converts the accumulated, unsustainable, potential IEM into kinetic IEM, eliciting the emergence of an *a priori* unpredictable organization that is more thermodynamically stable. Written another way, theory shows that the disassembled gyrosystem provides the architectural basis and thermodynamic driving force for evolutionary complexification. This theory supports what Gould and Eldredge implied in their theory of punctuated equilibrium [787]: evolutionary emergence occurs by a true quantal leap. I conclude that my theory is a comprehensive and scientifically accurate alternative to natural selection.

*Meaning of Life*. Life has many definitional meanings but lacks a complete and consistent scientific explanation. In this work, I have pursued and arrived at a scientific answer to the Schrödingerian question, “What is Life? [1].” Traditionally, the living cell is commonly called “animate” and all other biospheric and cellular chemicals and molecules are called “inanimate.” However, this theory and the law of vortex motion prove that all these physical systems gyrate and are, as such, “animated.” Moreover, theory-defined laws of unity and correspondence require that life and Earth evolve as one, with thermodynamically appropriate conditions (the fitness of the biosphere [788,789]). Unexpectedly, then, this theory reveals that Earth—or, for that matter, any celestial, physical, chemical, and molecular system—is alive, that is, synonymous with life. Given this definitional and conceptual upheaval, I propose that a very open and candid discussion of the meaning of life—well beyond this text—is in order. On this topic, it may be useful to consider how scientifically redefining life elucidates non-scientific, eudaemonic meanings of “life,” “living,” or “alive,” related to ontology, consciousness, sentience, behavior, vocation, or social interactions.

*Search for Extraterrestrial Life*. The quest to discover extraterrestrial forms of life in the universe is predicated on a set of definitions and assumptions of what life *is* [790]. Furthermore, astrobiologists and exobiologists seek an understanding of the conditions for habitability and distribution of life on other planets and throughout the cosmos [791,792,793]. In addition to modeling terrestrial, or Earth-bound, life, this general theory models exterrestrial forms of life. Indeed, this theory not only predicts but also proves that the universe is teeming with life—a result of omnidirectional universal evolution.

*Causality, Chance, and Necessity*. In the second half of the 20^th^ century, Monod painted a *Weltanschauung* in which life was happenstance, stochastic, and largely without purpose [794]. While this worldview may appeal to some, it was and is premature to conclude that it is the correct perspective of life. With this theory, I show that any measurement of the physical living system changes the system, causing it to evolve; any calculation or prediction excludes information. As gyrosystems model both the cause of a phenomenon *and* the effect of that phenomenon, it is accurate to write that the gyrosystem is the phenomenon itself. Therefore, cellular life is not chance, but necessity—by virtue of theoretically validated natural laws and of theoretically unified phenomena that have occurred in the evolutionary history of the universe and are occurring now, at every Cartesian coordinate throughout the universe.

*Metaphysics*. The gyre models the living universe *perfectly*. I have been unable to find one system, particle, event, or process—at any point or stage leading up to or during the origin of life—that does not consent to modeling onto the gyre form. In other words, there is no “before” or “after” the gyre in a spacetime sense; the gyre is evolutionarily and existentially omnipresent. This theory proves that the gyre is the long-sought invisible and inevitable metaphysical element of the universe, fulfilling a philosophical goal that dates to ancient Greece [795].

*Epistemological rupture*. The philosopher Bachelard claimed that scientific history is replete with unconsciously constructed or immanent “epistemological obstacles,” that are eventually broken through and shed during “epistemological rupture [796].” I conclude that my theoretical work elicits a Bachelardian rupture of intradisciplinary noöspheres and interdisciplinary boundaries. Kuhn proposed a related concept of “paradigm shift” to explain the process surrounding worldview conversion during a scientific revolution [797]. Whether the advent of this theory elicits a Kuhnian gestalt switch is debatable, though such an iconoclastic event has been foretold [798,799,800].

### 4.4. Concluding Remarks

In science and theory, the principle of parsimony dictates that the most straightforward, plain, and frugal model of an observation or set thereof is more favorable and likely right. As my theoretical framework coalesces a vast amount of accumulated scientific evidence into one neat, lawful, and interconnected modular structure, it abides by this principle. In conclusion, this catholic theory provides an innovative and elegant solution to the origin, evolution, and nature of life in the cosmos. I humbly proffer my theory as a viable system for knowing life.

## Supplementary Files

Supplementary File 1 Editorial: Publication of Controversial Papers in Life (Shu-Kun Lin) (PDF, 129 KB)
