# Supplementary material for: Theory of the Origin, Evolution, and Nature of Life
Source: Life (Basel). 2011 Dec 23;2(1):1–105. doi: 10.3390/life2010001 (PMC4187144; doi:10.3390/life2010001)
Supplement: Supplementary File 1 — Editorial: Publication of Controversial Papers in Life (Shu-Kun Lin) (PDF, 129 KB) [file life-02-00001-s001.pdf]

Life **2012**, *2*, 213-214; doi:10.3390/life2010213

OPEN ACCESS

life

ISSN 2075-1729
www.mdpi.com/journal/life

Editorial

Publication of Controversial Papers in *Life*

Shu-Kun Lin

MDPI AG, Postfach, CH-4005 Basel, Switzerland; E-Mail: lin@mdpi.com;
Website: http://www.mdpi.org/lin/

Received: 1 February 2012 / Accepted: 2 February 2012 / Published: 3 February 2012

Life (ISSN 2075-1729, http://www.mdpi.com/journal/life/) is a new journal that deals with new and sometime difficult interdisciplinary matters. Consequently, the journal will occasionally be presented with submitted articles that are controversial and/or outside conventional scientific views. Some papers recently accepted for publication in *Life* have attracted significant attention. Moreover, members of the Editorial Board have objected to these papers; some have resigned, and others have questioned the scientific validity of the contributions. In response I want to first state some basic facts regarding all publications in this journal. All papers are peer-reviewed, although it is often difficult to obtain expert reviewers for some of the interdisciplinary topics covered by this journal. I feel obliged to stress that although we will strive to guarantee the scientific standard of the papers published in this journal, all the responsibility for the ideas contained in the published articles rests entirely on their authors. Discussions on previously published articles are welcome and I hope that, by fostering discussion and by keeping an open-minded attitude towards new ideas, the journal will spur progress in this little explored, difficult and very exciting area of knowledge.

In particular, the paper "Andrulis, E.D. Theory of the Origin, Evolution, and Nature of Life. *Life* **2012**, *2*, 1-105" was published recently online and is due to appear in Issue 1, Volume 2, 2012 of *Life*, at the end of March this year [1]. So that our readership has as much information as I can divulge without violating the confidentiality of the review process, what follows is the background of these events. Professor Bassez had previously guest-edited a successful special issue titled "The Origin of Life" in another MDPI journal [2]. Although Professor Bassez [3] had also planned to be the Guest Editor of the special issue "Origin of Life - Feature Papers" for *Life* [4], she was, for personal reasons, unable to do so. I therefore volunteered to take this responsibility on her behalf and to guest edit this special issue and supervise the editorial procedure for the papers. I made the decision of acceptance based on the peer review reports we received and their recommendation in support of publication.

As stated earlier, finding reviewers able to cross discipline boundaries as is often needed for multidisciplinary "origin of life" topics [5] is particularly difficult. The publishing process that MDPI

manuscripts go through by our in-house editorial staff members is that they choose reviewers from sources like *Chemical Abstracts*, *PubMed*, *Web of Science* or more recently, from *Google Scholar*. Very often we also ask the Editorial Board members to review papers or ask those of them who have relevant knowledge and expertise to supply possible reviewer names. We also use the reviewer names suggested by the authors, but we do this with great care, checking the background of each potential reviewer and their publication record, as well as ensuring they have no collaborations with the authors that may be construed as a conflict of interest. I should stress that although we try to encourage bold, innovative science, we reject many submissions. In the case of the Dr. Andrulis's long paper, the two reviewers were both faculty members of reputable universities different than the author's and both went to considerable trouble presenting lengthy review reports. Dr. Andrulis revised his manuscript as requested, and the paper was subsequently published.

Regardless of opinion on specific papers that have been published to date, I sincerely hope that all of our articles, most of which are outstanding, will continue to be read and discussed. Our editorial procedure is under scrutiny by the Editorial Board, who wishes to be more closely involved in the editorial process, and we are striving to further improve our editorial service. We welcome comments on the Dr. Andrulis's paper or any other papers that have been published in *Life*.

References and Notes

1. See: http://www.mdpi.com/2075-1729/2/1/.
2. See: http://www.mdpi.com/journal/ijms/special_issues/origin-of-life/.
3. Bassez, M.-P. Emergence of Life. *Life* **2011**, *1*, 7-8; DOI:10.3390/life1010007.
4. See: http://www.mdpi.com/journal/life/special_issues/origin_life_feature/.
5. Lin, S.-K. Origin of Life and Birth of *Life*—An Open Access Journal. *Life* **2011**, *1*, 1-2; DOI:10.3390/life1010001.

© 2012 by the authors; licensee MDPI, Basel, Switzerland. This article is an open access article distributed under the terms and conditions of the Creative Commons Attribution license (http://creativecommons.org/licenses/by/3.0/).
